# Supplementary material for: The dynamics and longevity of circulating CD4+ memory T cells depend on cell age and not the chronological age of the host
Source: PLoS Biol. 2024 Aug 13;22(8):e3002380. doi: 10.1371/journal.pbio.3002380 (PMC11321570; doi:10.1371/journal.pbio.3002380)
Supplement: S1 File — (PDF) [file pbio.3002380.s001.pdf]

Supporting Information

The dynamics and longevity of circulating CD4$^+$ memory T cells depend on cell age and not the chronological age of the host

M. Elise Bullock[1]¤, Thea Hogan[2]¤, Cayman Williams[2]¤, Sinead Morris[1], Maria Nowicka[1], Minahil Sharjeel[2], Christiaan van Dorp[1], Andrew J. Yates[1]‡*, Benedict Seddon[2]‡*

[1] Department of Pathology and Cell Biology, Columbia University Irving Medical Center, 630 West 168th Street, New York, NY 10032, USA
[2] Institute of Immunity and Transplantation, Division of Infection and Immunity, UCL, Royal Free Hospital, Rowland Hill Street, London NW3 2PF, United Kingdom

¤ Contributed equally

‡ Contributed equally

*Address correspondence to either author; `andrew.yates@columbia.edu, benedict.seddon@ucl.ac.uk`

Supporting Tables

Population	Cohort	Daily influx (MAP and 95% CI) - cells per day	
		Donor	Host
CD4 T_{CM}	Young	1400 (1200, 1700)	610 (490, 740)
	Old	1400 (1200, 1600)	630 (520, 750)
CD4 T_{EM}	Young	14000 (12000, 16000)	8200 (6900, 9800)
	Old	13000 (11000, 15000)	9200 (7500, 11000)

Table A – MAP estimates and 95% credible intervals on the total daily rate of influx of cells into the CD4 T_{CM} and T_{EM} pools, relative to (Text C, eq. 1; and Fig C).

Age	Cohort	Population	Model	ELPD	SE
Old	Host	CD4 T_{CM}	Linear	0	N/A
			Branched	0.25	0.78
			Burst	3.86	4
Old	Host	CD4 T_{EM}	Branched	0	N/A
			Linear	0.27	0.9
			Burst	3.81	4.39
Old	Donor	CD4 T_{CM}	Linear	0	N/A
			Branched	0.43	0.62
			Burst	7.35	5.35
Old	Donor	CD4 T_{EM}	Branched	0	N/A
			Linear	2.34	0.85
			Burst	3.1	5.66
Young	Host	CD4 T_{CM}	Burst	0	N/A
			Linear	7.44	6.2
			Branched	7.89	5.99
Young	Host	CD4 T_{EM}	Burst	0	0N/A
			Branched	0.22	0.98
			Linear	1.71	0.46
Young	Donor	CD4 T_{CM}	Burst	0	N/A
			Branched	8.64	7.36
			Linear	9.39	7.47
Young	Donor	CD4 T_{EM}	Burst	0	N/A
			Linear	0.16	0.17
			Branched	0.18	1.36

Table B – Comparing support for the three models using the expected log pointwise predictive density (ELPD) with standard error (SE).

Parameter	Population	Cohort	Estimate (MAP and 95% CI)		
			Donor	Host	Difference
Fraction of influx entering fast subset	CM	Young	0.76 (0.52, 0.9)	0.61 (0.39, 0.88)	0.057 (-0.21, 0.38)
	CM	Old	0.55 (0.37, 0.8)	0.57 (0.34, 0.8)	0.028 (-0.32, 0.32)
	EM	Young	0.24 (0.15, 0.45)	0.24 (0.14, 0.53)	0.013 (-0.3, 0.23)
	EM	Old	0.31 (0.21, 0.56)	0.42 (0.28, 0.72)	-0.13 (-0.38, 0.17)
Mean interdivision time (Fast) (days)	CM	Young	4.8 (4.2, 5.4)	4.1 (3.5, 4.8)	0.64 (-0.19, 1.4)
	CM	Old	4.1 (3.6, 4.5)	3.8 (3.3, 4.4)	0.42 (-0.53, 0.99)
	EM	Young	7.5 (6.3, 8.7)	7.5 (5.9, 9.3)	0.26 (-2.3, 2.2)
	EM	Old	6.6 (5.6, 7.6)	6.2 (4.8, 8.4)	0.58 (-2, 2.2)
Mean lifetime (Fast) (days)	CM	Young	4.5 (4, 5)	3.9 (3.4, 4.5)	0.51 (-0.26, 1.2)
	CM	Old	3.8 (3.4, 4.3)	3.5 (3, 4.2)	0.43 (-0.51, 0.96)
	EM	Young	6.8 (5.9, 7.9)	6.7 (5.3, 8.6)	0.2 (-2, 1.9)
	EM	Old	5.8 (4.9, 6.7)	4.9 (3.7, 5.9)	1.1 (-0.37, 2.6)
Mean interdivision time (Slow) (days)	CM	Young	180 (98, 750)	190 (130, 510)	3.4 (-220, 560)
	CM	Old	140 (93, 360)	150 (110, 450)	-6.4 (-280, 190)
	EM	Young	340 (190, 900)	160 (110, 460)	200 (-100, 750)
	EM	Old	130 (93, 330)	140 (110, 360)	-12 (-170, 170)
Mean lifetime (Slow) (days)	CM	Young	56 (43, 95)	120 (98, 160)	-56 (-99, -20)
	CM	Old	61 (55, 73)	71 (62, 87)	-9.1 (-26, 5.1)
	EM	Young	40 (36, 50)	72 (62, 86)	-29 (-45, -18)
	EM	Old	47 (42, 55)	57 (52, 68)	-10 (-21, -1.4)
Efficiency of BrdU uptake per division	CM	Young	0.53 (0.51, 0.57)	0.54 (0.52, 0.58)	-0.011 (-0.053, 0.039)
	CM	Old	0.66 (0.63, 0.7)	0.66 (0.61, 0.71)	-0.0027 (-0.063, 0.064)
	EM	Young	0.56 (0.51, 0.6)	0.57 (0.52, 0.62)	-0.0047 (-0.077, 0.064)
	EM	Old	0.73 (0.68, 0.78)	0.69 (0.64, 0.77)	0.043 (-0.055, 0.12)
Rate of loss of BrdU in source during delabelling	CM	Young	0.18 (0.15, 0.21)	0.17 (0.15, 0.2)	0.0059 (-0.032, 0.04)
	CM	Old	0.18 (0.15, 0.2)	0.17 (0.15, 0.2)	0.0038 (-0.036, 0.041)
	EM	Young	0.19 (0.16, 0.22)	0.18 (0.15, 0.21)	-0.0018 (-0.042, 0.049)
	EM	Old	0.19 (0.16, 0.22)	0.18 (0.16, 0.22)	-0.0014 (-0.043, 0.046)
Ki67 lifetime (days)	CM	Young	3.1 (3.1, 3.2)	3.1 (3.1, 3.2)	0.0041 (-0.07, 0.074)
	CM	Old	3.2 (3.1, 3.3)	3.2 (3.1, 3.4)	-0.0062 (-0.21, 0.12)
	EM	Young	3.1 (3.1, 3.2)	3.1 (3.1, 3.2)	0.0005 (-0.068, 0.057)
	EM	Old	3.1 (3.1, 3.2)	3.2 (3.1, 3.3)	-0.021 (-0.15, 0.056)
Proportion Fast	CM	Young	0.69 (0.65, 0.75)	0.39 (0.35, 0.42)	0.31 (0.24, 0.37)
	CM	Old	0.44 (0.4, 0.47)	0.37 (0.34, 0.41)	0.062 (0.0082, 0.11)
	EM	Young	0.43 (0.38, 0.49)	0.21 (0.16, 0.28)	0.21 (0.13, 0.29)
	EM	Old	0.3 (0.25, 0.34)	0.16 (0.13, 0.2)	0.13 (0.076, 0.19)
Net loss rate of fast cells (λ_A)	CM	Young	0.014 (0.0096, 0.018)	0.0092 (0.0053, 0.015)	0.0042 (-0.0017, 0.01)
	CM	Old	0.014 (0.0099, 0.025)	0.016 (0.009, 0.028)	-0.00091 (-0.013, 0.011)
	EM	Young	0.0095 (0.0059, 0.019)	0.011 (0.0051, 0.025)	0.00072 (-0.016, 0.0097)
	EM	Old	0.016 (0.011, 0.032)	0.054 (0.028, 0.093)	-0.031 (-0.072, -0.0044)
Net loss rate of slow cells (λ_B)	CM	Young	0.012 (0.0048, 0.019)	0.0031 (0.0011, 0.006)	0.0091 (0.0012, 0.016)
	CM	Old	0.01 (0.0045, 0.014)	0.0087 (0.0035, 0.012)	0.0025 (-0.0052, 0.0082)
	EM	Young	0.021 (0.017, 0.026)	0.0076 (0.005, 0.011)	0.013 (0.0083, 0.018)
	EM	Old	0.015 (0.0092, 0.019)	0.012 (0.0061, 0.015)	0.003 (-0.0033, 0.01)
Ratio of fast:slow loss rates (λ_A/λ_B)	CM	Young	0.97 (0.53, 3.4)	2.2 (1, 12)	-1.1 (-11, 1.2)
	CM	Old	1.5 (0.78, 5.1)	2 (0.84, 7)	-0.33 (-5.3, 3.5)
	EM	Young	0.41 (0.26, 1)	1.2 (0.53, 4.8)	-0.66 (-3.9, 0.18)
	EM	Old	1.1 (0.65, 3.3)	4.2 (1.9, 15)	-2.7 (-13, 0.14)
Proportion Ki67^{high}	CM	Young	0.53 (0.51, 0.54)	0.32 (0.31, 0.34)	0.2 (0.18, 0.23)
	CM	Old	0.38 (0.36, 0.39)	0.34 (0.32, 0.35)	0.039 (0.017, 0.062)
	EM	Young	0.29 (0.27, 0.31)	0.17 (0.16, 0.18)	0.12 (0.1, 0.14)
	EM	Old	0.24 (0.23, 0.25)	0.17 (0.16, 0.18)	0.073 (0.06, 0.089)
Clonal half-life of slow (days)	CM	Young	50 (36, 140)	170 (110, 600)	-120 (-560, -37)
	CM	Old	67 (50, 150)	79 (56, 200)	-10 (-130, 72)
	EM	Young	32 (27, 41)	82 (64, 140)	-50 (-110, -30)
	EM	Old	46 (36, 75)	61 (45, 110)	-12 (-63, 17)

Table C – MAP estimates and 95% credible intervals on parameters estimated using the branched model.

Parameter	Population	Cohort	Estimate (MAP and 95% CI)		
			Donor	Host	Difference
Rate of fast to slow	CM	Young	0.0028 (0.0015, 0.0097)	0.0041 (0.0018, 0.01)	0.00033 (-0.0068, 0.0052)
	CM	Old	0.0056 (0.0019, 0.018)	0.0048 (0.0018, 0.018)	0.00078 (-0.012, 0.012)
	EM	Young	0.0071 (0.0024, 0.028)	0.0059 (0.0028, 0.028)	0.00082 (-0.019, 0.021)
	EM	Old	0.026 (0.01, 0.033)	0.036 (0.012, 0.053)	-0.01 (-0.035, 0.016)
Mean interdivision time(Fast) (days)	CM	Young	4.9 (4.2, 5.4)	4.1 (3.5, 4.8)	0.71 (-0.19, 1.6)
	CM	Old	4 (3.6, 4.6)	3.7 (3.2, 4.4)	0.32 (-0.55, 1.1)
	EM	Young	7.9 (6.7, 11)	7.3 (5.8, 10)	0.51 (-2.6, 3.7)
	EM	Old	6.3 (5.3, 7.5)	5.8 (4.7, 7.5)	0.61 (-1.4, 2)
Mean lifetime (Fast) (days)	CM	Young	4.6 (3.9, 5)	3.9 (3.4, 4.6)	0.55 (-0.24, 1.3)
	CM	Old	3.8 (3.3, 4.3)	3.4 (3, 4.1)	0.31 (-0.55, 1)
	EM	Young	7.8 (6.1, 9.1)	6.9 (5, 8.6)	1.6 (-1.7, 3.1)
	EM	Old	6 (5.1, 7.1)	5.1 (4.2, 6.7)	0.64 (-0.9, 2.2)
Mean interdivision time (Slow) (days)	CM	Young	140 (81, 550)	160 (120, 390)	-20 (-210, 390)
	CM	Old	97 (78, 220)	100 (84, 220)	-7.8 (-110, 110)
	EM	Young	120 (64, 730)	91 (77, 140)	20 (-45, 630)
	EM	Old	94 (74, 140)	100 (87, 170)	-8 (-69, 36)
Mean lifetime (Slow) (days)	CM	Young	52 (40, 100)	110 (93, 150)	-55 (-93, -4.8)
	CM	Old	63 (51, 77)	74 (60, 95)	-13 (-35, 8.9)
	EM	Young	46 (36, 110)	73 (64, 95)	-31 (-49, 29)
	EM	Old	49 (44, 60)	62 (55, 77)	-15 (-28, -0.32)
Efficiency of BrdU uptake per division	CM	Young	0.53 (0.51, 0.57)	0.54 (0.51, 0.59)	-0.0013 (-0.059, 0.038)
	CM	Old	0.67 (0.63, 0.71)	0.68 (0.63, 0.73)	-0.011 (-0.069, 0.062)
	EM	Young	0.58 (0.53, 0.64)	0.61 (0.56, 0.66)	-0.019 (-0.099, 0.056)
	EM	Old	0.77 (0.72, 0.81)	0.76 (0.7, 0.81)	0.0067 (-0.066, 0.089)
Rate of loss of BrdU in source during delabelling	CM	Young	0.17 (0.15, 0.21)	0.17 (0.15, 0.2)	0.0056 (-0.032, 0.043)
	CM	Old	0.17 (0.15, 0.2)	0.17 (0.15, 0.2)	0.0051 (-0.034, 0.042)
	EM	Young	0.19 (0.16, 0.22)	0.18 (0.15, 0.21)	-0.0024 (-0.036, 0.046)
	EM	Old	0.17 (0.15, 0.21)	0.18 (0.15, 0.21)	-0.0085 (-0.043, 0.039)
Ki67 lifetime (days)	CM	Young	3.1 (3.1, 3.2)	3.1 (3.1, 3.2)	-0.0034 (-0.087, 0.059)
	CM	Old	3.1 (3.1, 3.3)	3.2 (3.1, 3.4)	-0.0091 (-0.25, 0.11)
	EM	Young	3.1 (3.1, 3.2)	3.1 (3.1, 3.2)	-0.00099 (-0.066, 0.046)
	EM	Old	3.1 (3.1, 3.2)	3.2 (3.1, 3.3)	-0.0098 (-0.14, 0.045)
Proportion Fast	CM	Young	0.72 (0.65, 0.75)	0.38 (0.35, 0.42)	0.32 (0.25, 0.38)
	CM	Old	0.43 (0.4, 0.47)	0.37 (0.33, 0.41)	0.067 (0.008, 0.11)
	EM	Young	0.51 (0.38, 0.62)	0.21 (0.15, 0.27)	0.31 (0.15, 0.42)
	EM	Old	0.29 (0.25, 0.35)	0.16 (0.14, 0.21)	0.13 (0.07, 0.19)
Net loss rate of fast cells (λ_A)	CM	Young	0.013 (0.0085, 0.019)	0.01 (0.0038, 0.015)	0.0019 (-0.0036, 0.012)
	CM	Old	0.019 (0.0067, 0.027)	0.019 (0.0086, 0.03)	-0.0027 (-0.017, 0.013)
	EM	Young	0.017 (0.0025, 0.027)	0.023 (0.0049, 0.041)	-0.011 (-0.03, 0.014)
	EM	Old	0.003 (0.00047, 0.021)	0.0092 (0.0027, 0.047)	-0.0051 (-0.042, 0.0092)
Net loss rate of slow cells (λ_B)	CM	Young	0.014 (0.0038, 0.02)	0.0025 (0.0011, 0.0065)	0.011 (0.000059, 0.017)
	CM	Old	0.0044 (0.0014, 0.014)	0.0029 (0.00094, 0.011)	0.0017 (-0.0063, 0.01)
	EM	Young	0.0078 (0.003, 0.024)	0.0016 (0.00063, 0.0072)	0.0051 (-0.0012, 0.022)
	EM	Old	0.011 (0.0043, 0.014)	0.0071 (0.0022, 0.011)	0.0027 (-0.0039, 0.0096)
Ratio of fast:slow loss rates (λ_A/λ_B)	CM	Young	0.88 (0.44, 4.7)	2.3 (0.64, 12)	-1.1 (-9.7, 2.4)
	CM	Old	2.1 (0.51, 18)	3.7 (0.88, 28)	-1.3 (-24, 13)
	EM	Young	0.87 (0.099, 8.1)	6.3 (0.7, 58)	-4.7 (-55, 3.4)
	EM	Old	0.4 (0.034, 4.8)	2.1 (0.25, 22)	-1.3 (-21, 2.5)
Proportion Ki67^{high}	CM	Young	0.53 (0.51, 0.55)	0.32 (0.3, 0.34)	0.2 (0.18, 0.23)
	CM	Old	0.38 (0.36, 0.39)	0.33 (0.32, 0.36)	0.037 (0.016, 0.061)
	EM	Young	0.3 (0.28, 0.32)	0.17 (0.16, 0.18)	0.13 (0.11, 0.16)
	EM	Old	0.24 (0.23, 0.26)	0.17 (0.16, 0.18)	0.075 (0.059, 0.089)
Clonal half-life of slow (days)	CM	Young	49 (35, 180)	180 (110, 620)	-130 (-540, -0.86)
	CM	Old	91 (49, 490)	140 (64, 740)	-43 (-610, 290)
	EM	Young	48 (29, 230)	210 (97, 1100)	-140 (-1100, 43)
	EM	Old	64 (50, 160)	90 (62, 320)	-23 (-250, 56)

Table D – MAP estimates and 95% credible intervals on parameters estimated using the linear model.

Parameter	Population	Cohort	Estimate (MAP and 95% CI)		
			Donor	Host	Difference
Rate of fast to slow	CM	Young	0.0051 (0.0032, 0.0096)	0.01 (0.0077, 0.016)	-0.0045 (-0.011, 0.00033)
	CM	Old	0.021 (0.015, 0.027)	0.024 (0.017, 0.032)	-0.0023 (-0.012, 0.006)
	EM	Young	0.0097 (0.0027, 0.021)	0.02 (0.0099, 0.04)	-0.011 (-0.03, 0.0041)
	EM	Old	0.037 (0.026, 0.05)	0.063 (0.044, 0.086)	-0.025 (-0.053, -0.0028)
Mean interdivision time (Fast) (days)	CM	Young	4.4 (3.8, 4.7)	3.8 (3.3, 4.3)	0.58 (-0.22, 1.1)
	CM	Old	4 (3.7, 4.6)	3.9 (3.3, 4.5)	0.099 (-0.65, 0.92)
	EM	Young	7.4 (6.3, 8.8)	7.9 (6.6, 9.7)	-0.24 (-2.4, 1.5)
	EM	Old	6.6 (5.9, 7.5)	6.9 (5.4, 8.2)	0.23 (-1.7, 1.5)
Mean lifetime (Fast) (days)	CM	Young	4.1 (3.6, 4.5)	3.6 (3.2, 4.1)	0.52 (-0.29, 0.99)
	CM	Old	3.8 (3.4, 4.2)	3.6 (3.1, 4.2)	0.14 (-0.56, 0.81)
	EM	Young	6.7 (6, 7.8)	7.5 (6.2, 9)	-0.61 (-2.3, 1)
	EM	Old	6.3 (5.5, 7.2)	6.1 (4.8, 7.3)	0.31 (-1.2, 1.8)
Mean interdivision time (Slow) (days)	CM	Young	150 (100, 350)	280 (200, 370)	-130 (-230, 83)
	CM	Old	120 (98, 170)	150 (110, 170)	-17 (-53, 34)
	EM	Young	340 (140, 1300)	220 (140, 970)	98 (-480, 1000)
	EM	Old	120 (88, 220)	110 (97, 210)	-15 (-78, 90)
Mean lifetime (Slow) (days)	CM	Young	84 (49, 140)	190 (130, 270)	-100 (-200, -32)
	CM	Old	88 (68, 120)	100 (80, 130)	-14 (-54, 22)
	EM	Young	61 (41, 230)	96 (77, 190)	-43 (-120, 120)
	EM	Old	57 (50, 80)	73 (63, 99)	-15 (-42, 5.8)
Efficiency of BrdU uptake per division	CM	Young	0.51 (0.48, 0.54)	0.51 (0.48, 0.55)	-0.005 (-0.053, 0.046)
	CM	Old	0.65 (0.61, 0.68)	0.65 (0.61, 0.7)	-0.0058 (-0.062, 0.051)
	EM	Young	0.52 (0.49, 0.57)	0.5 (0.47, 0.56)	0.018 (-0.035, 0.077)
	EM	Old	0.71 (0.67, 0.76)	0.7 (0.64, 0.75)	0.031 (-0.056, 0.086)
Rate of loss of BrdU in source during delabeling	CM	Young	0.17 (0.14, 0.2)	0.16 (0.14, 0.2)	0.0041 (-0.035, 0.039)
	CM	Old	0.16 (0.14, 0.19)	0.16 (0.14, 0.2)	-0.005 (-0.037, 0.037)
	EM	Young	0.18 (0.15, 0.2)	0.17 (0.15, 0.21)	0.0022 (-0.038, 0.043)
	EM	Old	0.17 (0.15, 0.21)	0.17 (0.15, 0.2)	0.0057 (-0.041, 0.04)
Ki67 lifetime (days)	CM	Young	2.3 (2.1, 2.4)	2.2 (2, 2.4)	0.052 (-0.23, 0.31)
	CM	Old	2.7 (2.5, 3)	2.8 (2.6, 3.1)	-0.071 (-0.47, 0.28)
	EM	Young	1.9 (1.8, 2.1)	2 (1.8, 2.1)	-0.0011 (-0.2, 0.19)
	EM	Old	2.5 (2.3, 2.7)	2.7 (2.4, 2.9)	-0.16 (-0.48, 0.21)
Proportion Fast	CM	Young	0.76 (0.72, 0.8)	0.44 (0.41, 0.48)	0.31 (0.26, 0.37)
	CM	Old	0.48 (0.45, 0.51)	0.41 (0.38, 0.45)	0.052 (0.018, 0.11)
	EM	Young	0.63 (0.56, 0.72)	0.38 (0.31, 0.44)	0.26 (0.15, 0.37)
	EM	Old	0.4 (0.35, 0.44)	0.25 (0.21, 0.29)	0.16 (0.086, 0.21)
Net loss rate of fast cells (λ_A)	CM	Young	0.014 (0.0092, 0.021)	0.011 (0.0066, 0.016)	0.0037 (-0.0039, 0.01)
	CM	Old	0.018 (0.012, 0.029)	0.018 (0.0094, 0.031)	0.00022 (-0.013, 0.014)
	EM	Young	0.013 (0.0029, 0.019)	0.008 (0.00087, 0.016)	-0.00024 (-0.0073, 0.014)
	EM	Old	0.0053 (0.001, 0.02)	0.011 (0.0027, 0.038)	-0.0057 (-0.031, 0.0086)
Net loss rate of slow cells (λ_B)	CM	Young	0.0056 (0.00064, 0.016)	0.00082 (0.00012, 0.0045)	0.0055 (-0.0011, 0.014)
	CM	Old	0.0012 (0.00024, 0.0079)	0.0011 (0.00022, 0.0061)	-0.000053 (-0.0038, 0.006)
	EM	Young	0.011 (0.0012, 0.023)	0.0073 (0.0007, 0.01)	0.0075 (-0.0057, 0.018)
	EM	Old	0.011 (0.0022, 0.014)	0.0072 (0.00064, 0.01)	0.0041 (-0.0044, 0.011)
Ratio of fast:slow loss rates (λ_A/λ_B)	CM	Young	3.3 (0.68, 27)	8.7 (1.8, 110)	-5.2 (-100, 17)
	CM	Old	8.9 (1.6, 92)	17 (1.7, 96)	-1.7 (-85, 82)
	EM	Young	1.8 (0.15, 14)	3 (0.096, 24)	-0.51 (-20, 13)
	EM	Old	1.2 (0.082, 8.8)	9.9 (0.28, 51)	-6.1 (-46, 6.1)
Proportion Ki67^{high}	CM	Young	0.5 (0.48, 0.52)	0.31 (0.3, 0.33)	0.19 (0.17, 0.22)
	CM	Old	0.37 (0.36, 0.39)	0.33 (0.32, 0.35)	0.038 (0.014, 0.061)
	EM	Young	0.27 (0.26, 0.29)	0.16 (0.15, 0.17)	0.11 (0.095, 0.13)
	EM	Old	0.23 (0.22, 0.25)	0.17 (0.16, 0.18)	0.071 (0.054, 0.084)
Clonal half-life of slow (days)	CM	Young	160 (45, 1100)	510 (160, 5700)	-340 (-5500, 530)
	CM	Old	300 (88, 2900)	470 (110, 3200)	-72 (-2900, 2400)
	EM	Young	94 (30, 570)	190 (68, 990)	-96 (-940, 470)
	EM	Old	88 (49, 320)	210 (66, 1100)	-120 (-920, 210)

Table E – MAP estimates and 95% credible intervals on parameters estimated using the burst model.

Supporting Figures

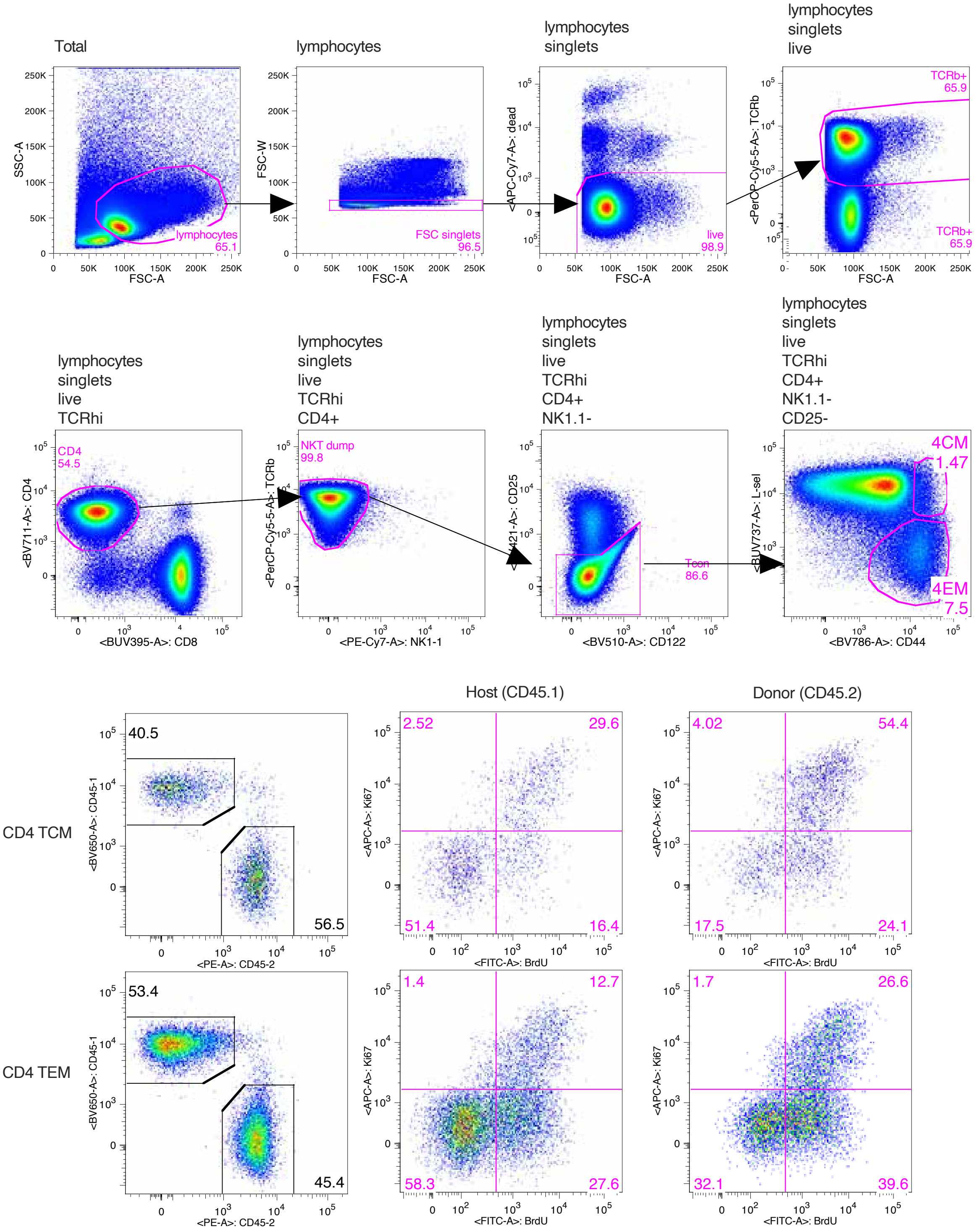

Figure A – Complete gating strategy for identifying host and donor CD4 T_{CM} and T_{EM}.

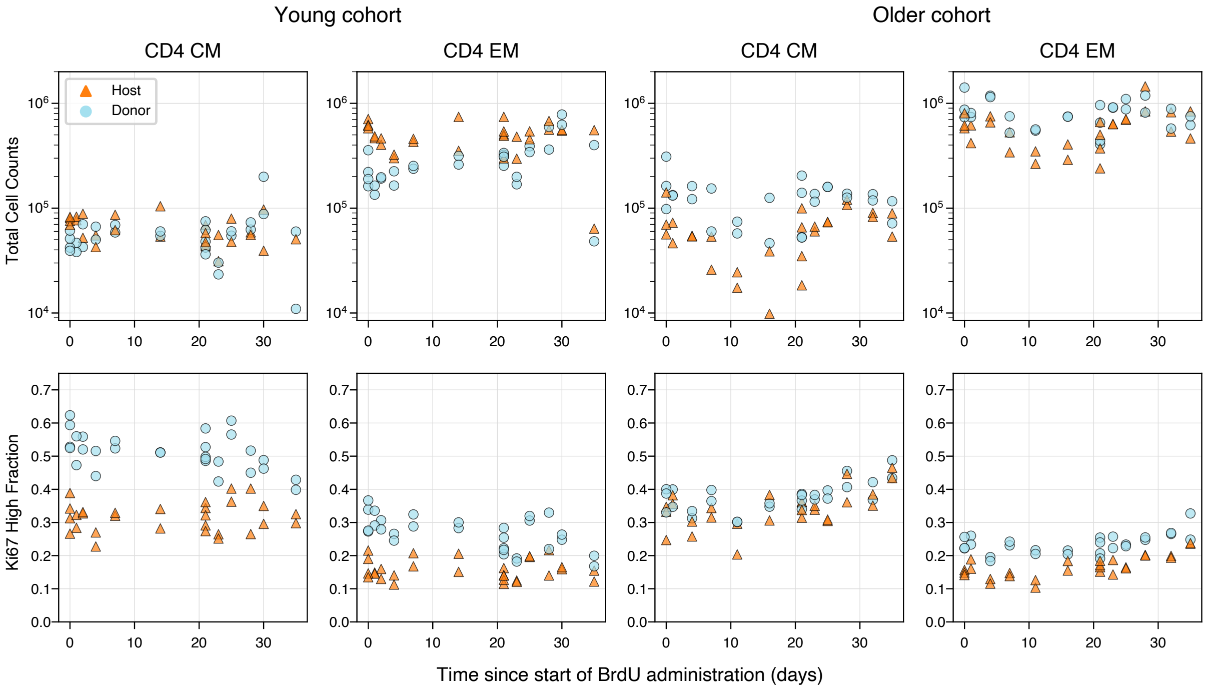

Figure B – Numbers and Ki67 expression of both host and donor CD4 T_{CM} and T_{EM} over the timecourses of the labelling experiments in young and older cohorts of busulfan chimeras. The data underlying the graphs shown in the figure can be found in S1 Data.

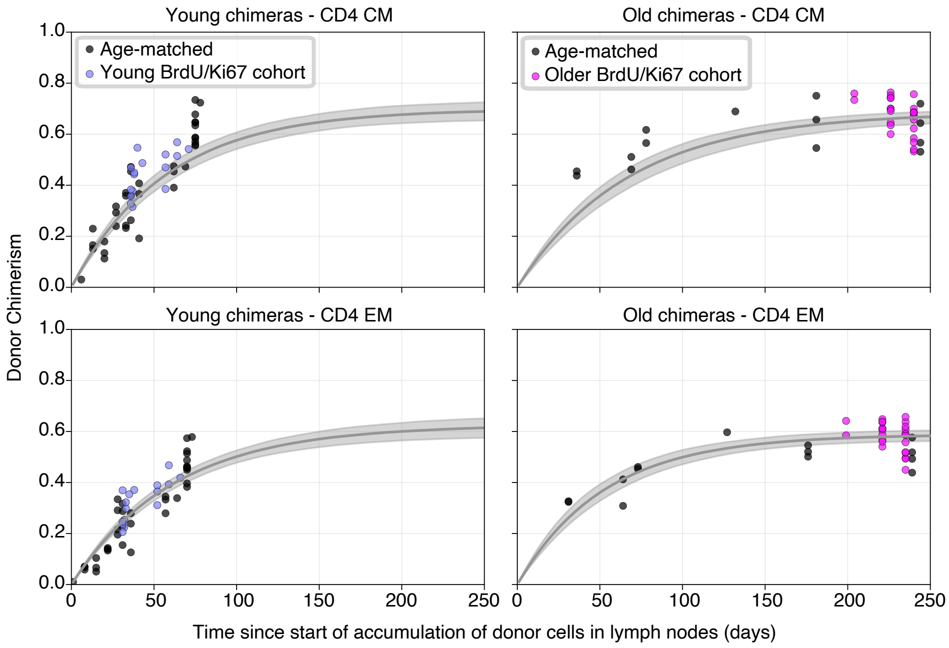

Figure C – **Estimating the influx into the CD4 T_{CM} and T_{EM} pools in young and old mice**. Curves are the fitted trajectories of $D(t)/N$, from the solution to Eq 1 in Text C. Black points represent data from age-matched busulfan chimeric mice from other experiments. Envelopes are derived from sampling the 95% credible intervals of the parameters, and indicate where 95% of the resulting trajectories lie. The data underlying the graphs shown in the figure can be found in S1 Data.

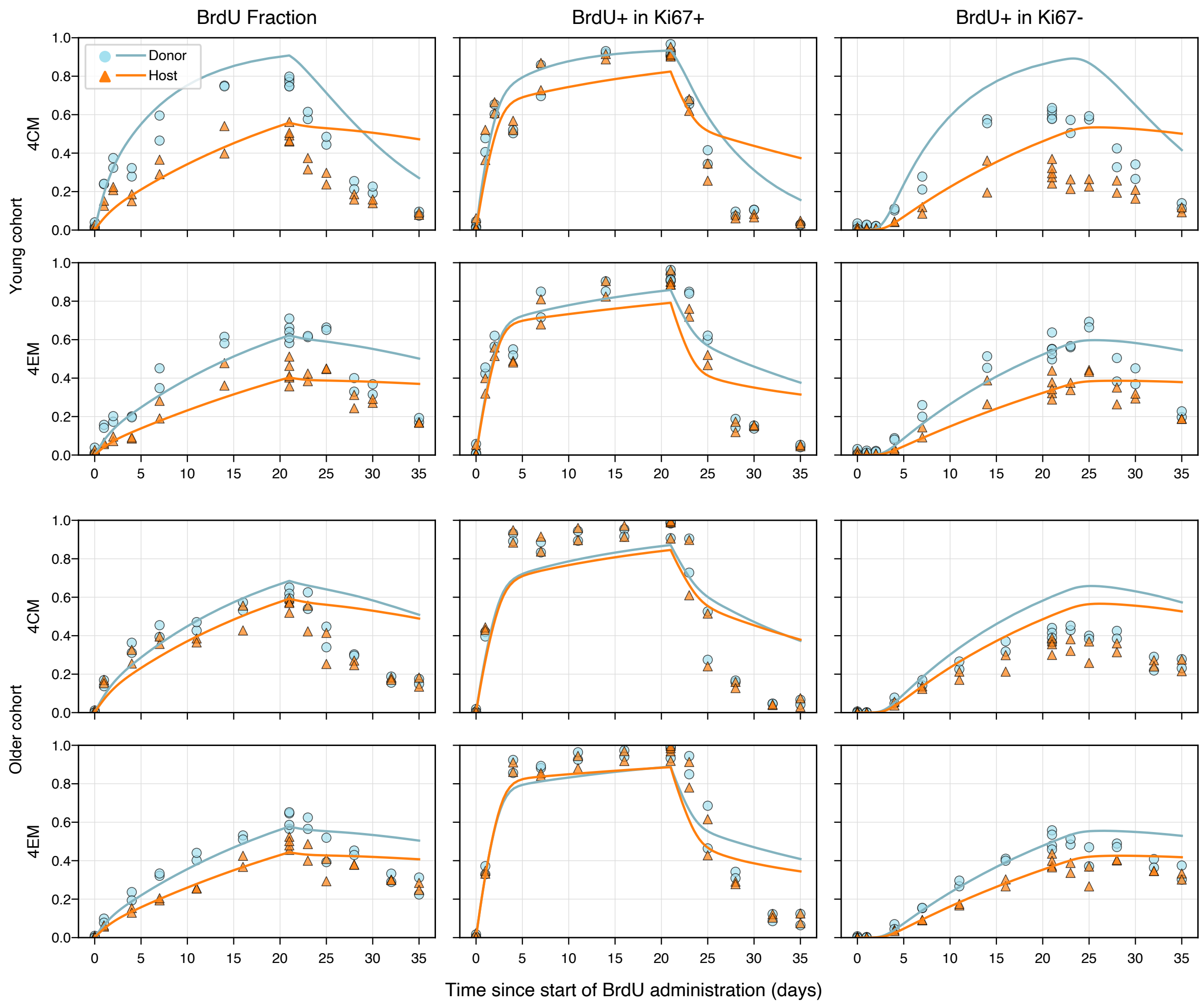

Figure D – Fits of the temporal heterogeneity model to the BrdU/Ki67 labelling data. The data underlying the graphs shown in the figure can be found in S1 Data.

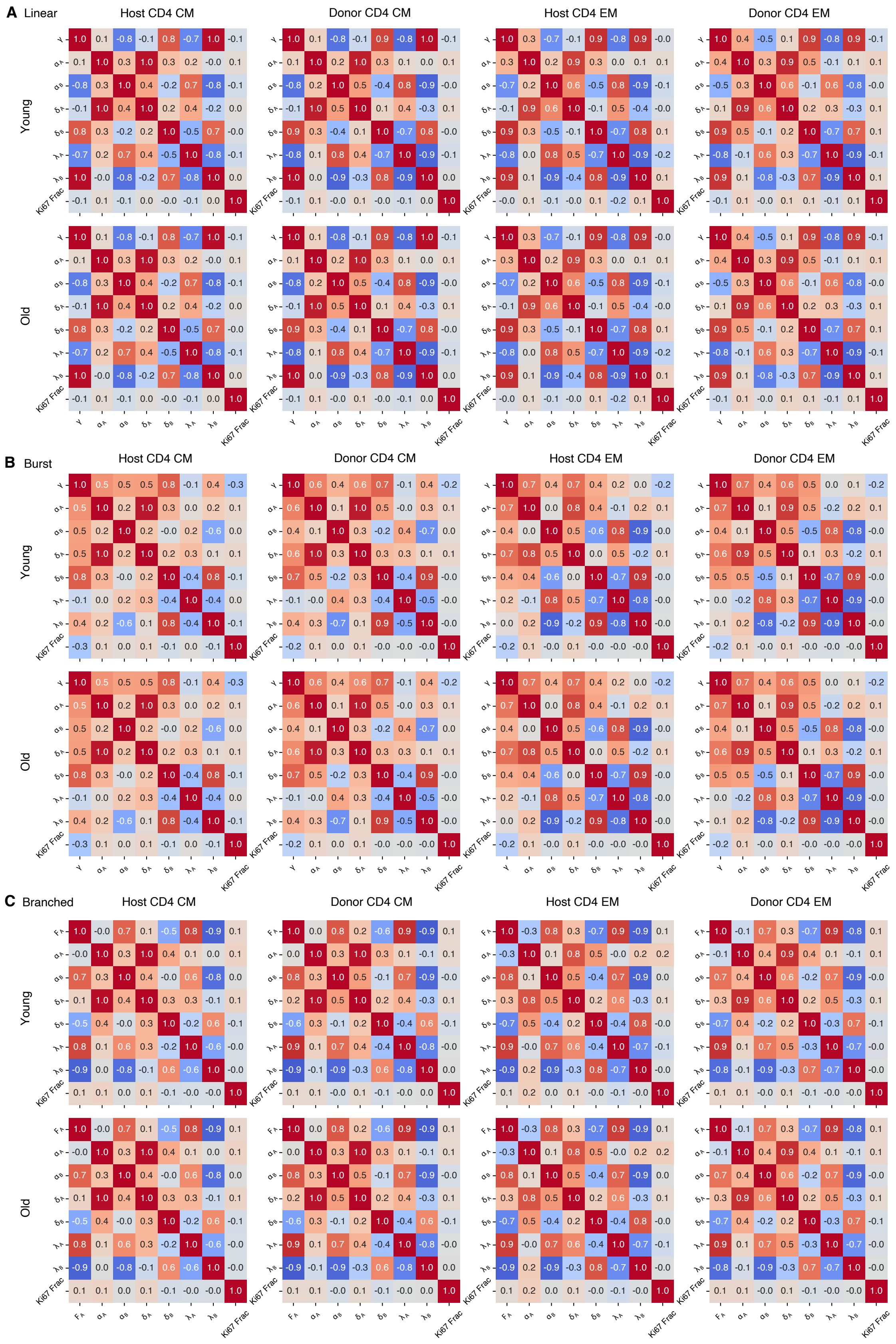

Figure E – Correlations in parameters in the fits of the linear model (A), the branched model (B) and the burst model (C).

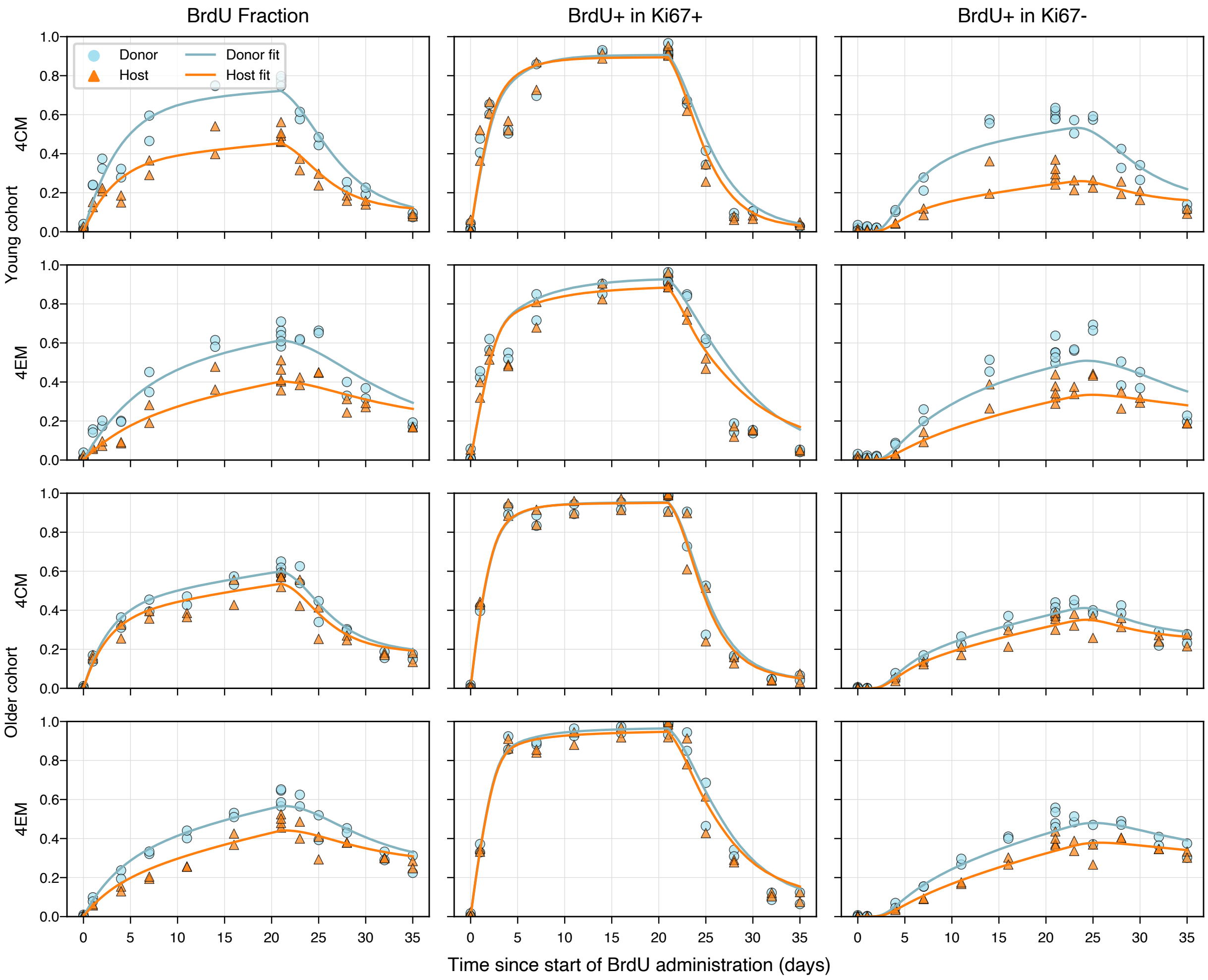

Figure F – Fits of the linear model to the BrdU/Ki67 labelling data. The data underlying the graphs shown in the figure can be found in S1 Data.

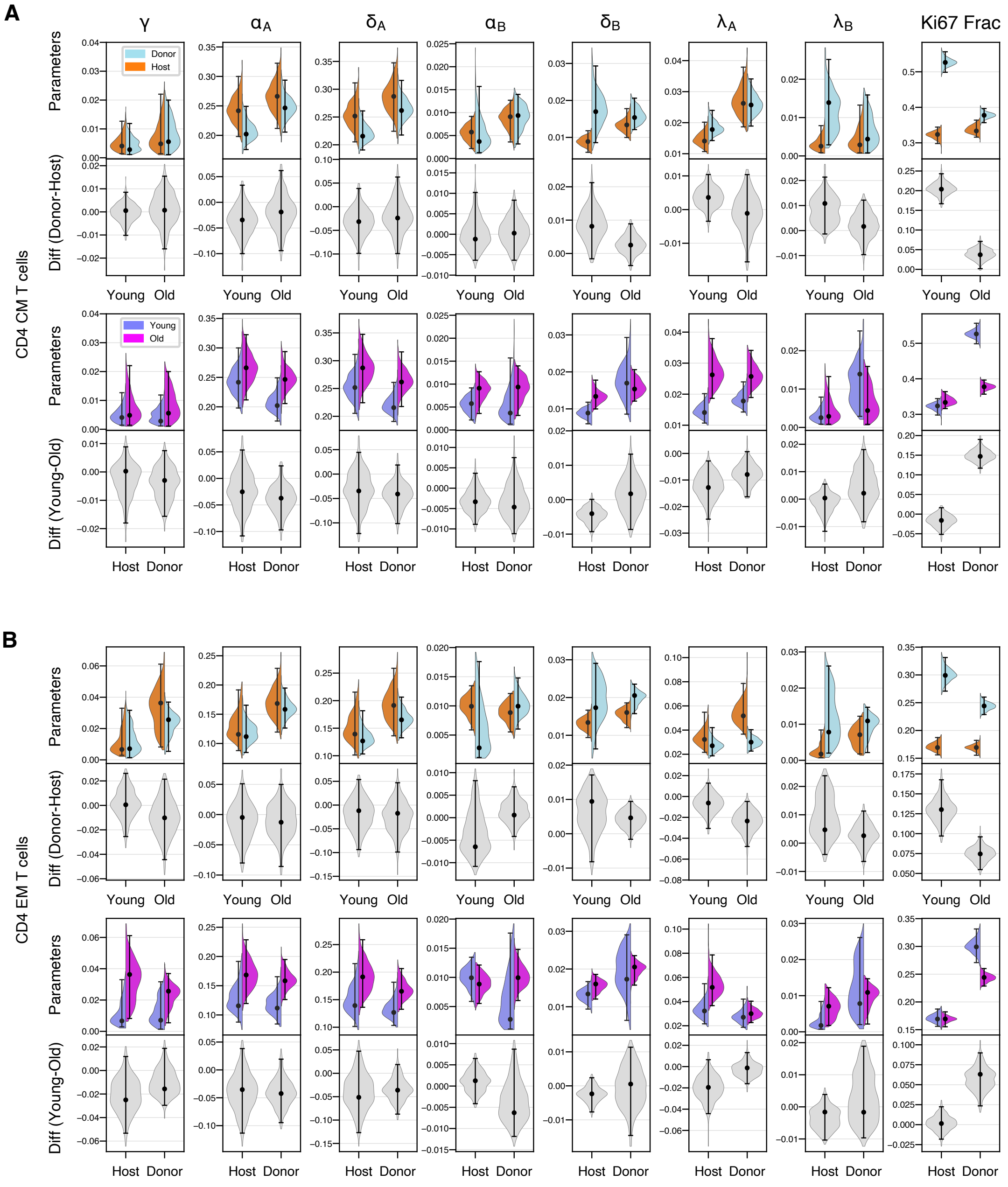

Figure G – Parameter estimates for the linear model, for CD4 T_{CM} (**A**) and CD4 T_{EM} (**B**). The data underlying the graphs shown in the figure can be found in S1 Data.

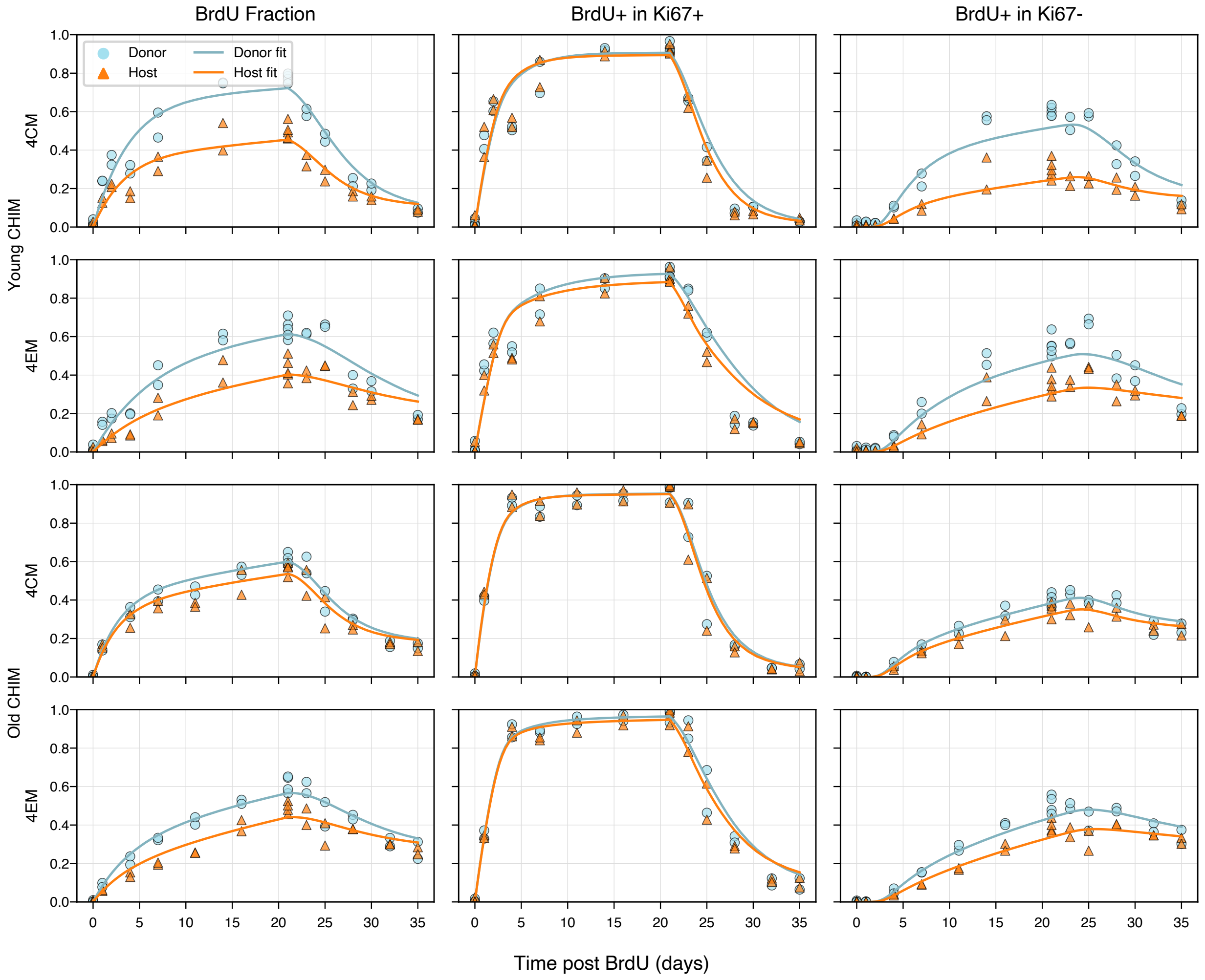

Figure H – Fits of the burst model to the BrdU/Ki67 labelling data. The data underlying the graphs shown in the figure can be found in S1 Data.

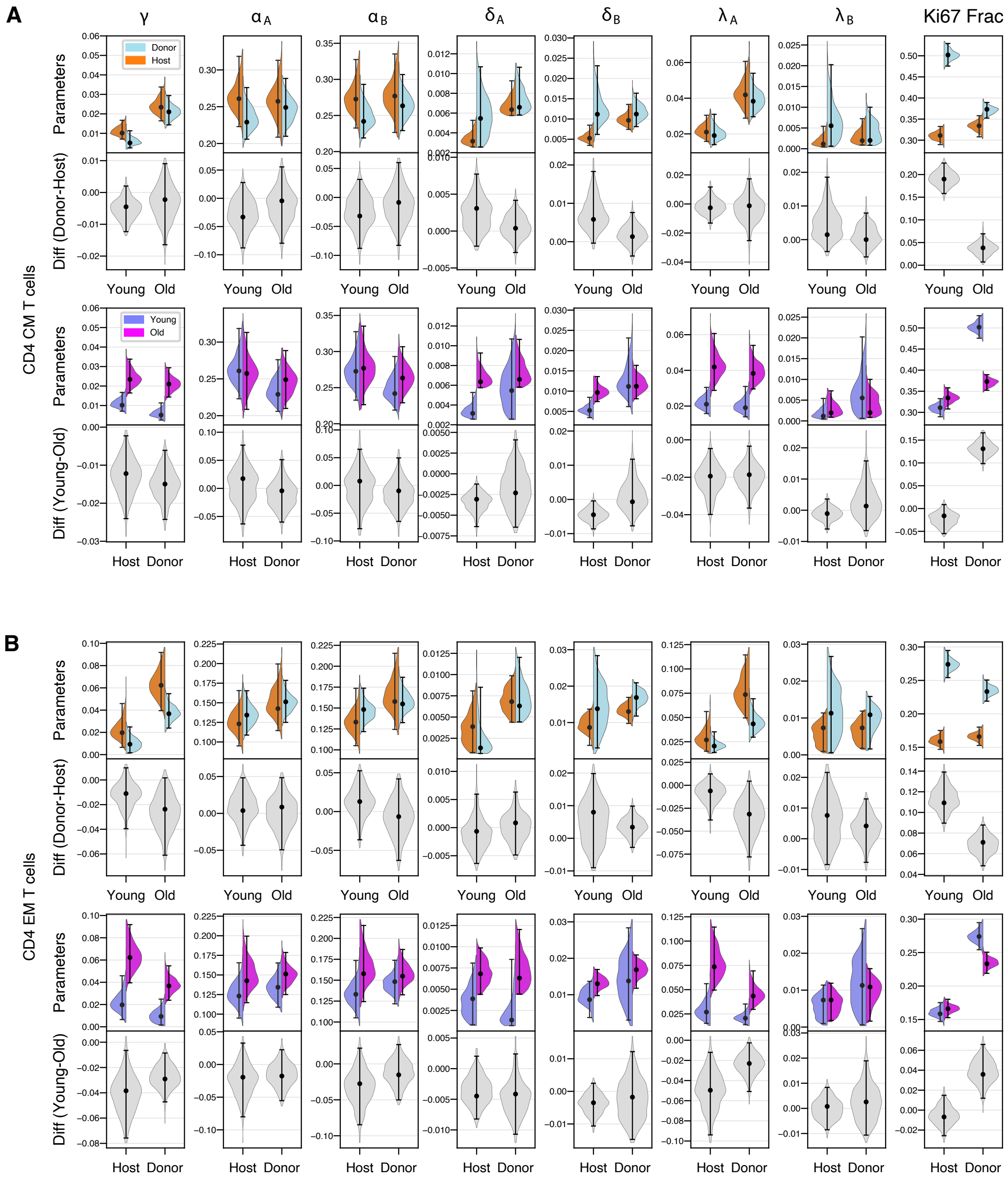

Figure I – Parameter estimates for the burst model, for CD4 T_{CM} (**A**) and CD4 T_{EM} (**B**). The data underlying the graphs shown in the figure can be found in S1 Data.

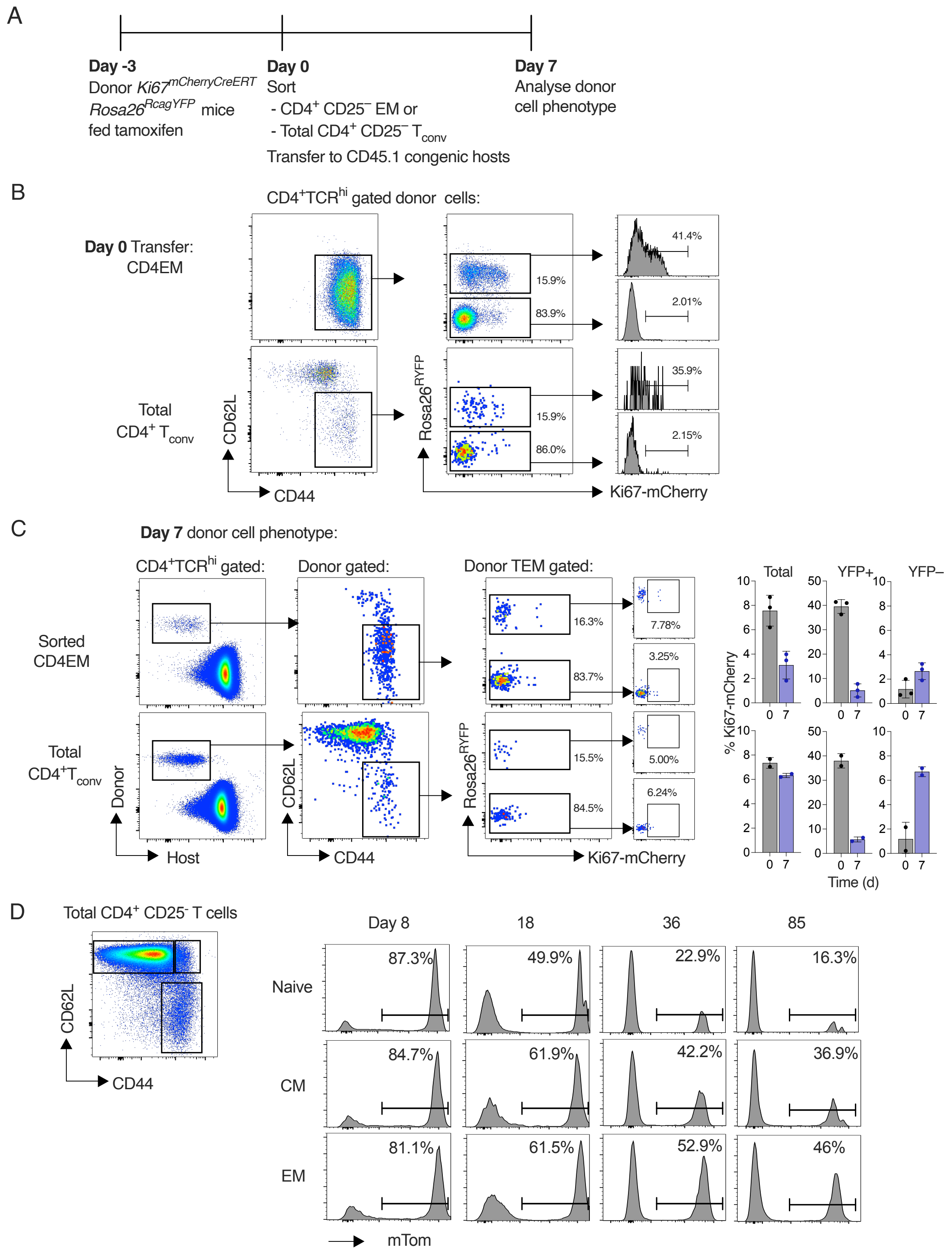

Figure J – Fate mapping Ki67-expressing and CD4-expressing cells using reporter mice. (A) Ki67$^{mCherry-CreERT}$ Rosa26^{RcagYFP} donors were fed one dose of tamoxifen (day -3) . Three days later, CD4 T_{EM} or conventional CD4 T cells in bulk were purified and transferred to CD45.1 congenic hosts (day 0). At day 7, LN were recovered from hosts and donor cell phenotype analysed by flow cytometry. (B) Density plots show naive (CD44^{lo} CD62^{hi} vs. T_{EM} composition of purified populations prior to transfer, and Ki67-mcherry expression by YFP$^+$ and YFP$^-$ in both donor populations prior to transfer. (C) Analysis of CD45.2$^+$ CD4 T_{EM} donor populations for expression of YFP and Ki67-mCherry. Data are from three independent replicates. (D) CD4^{CreERT} Rosa26^{RmTom} reporter mice were injected with tamoxifen and culled at different days following treatment. Plots show gating strategy to identify CD4$^+$ naive, T_{CM} and T_{EM} populations and examples of mTom reporter expression by these subsets at different times after tamoxifen injection. The data underlying the graphs shown in the figure can be found in S1 Data.

Text A Mathematical modelling

Modelling BrdU/Ki67 pulse-chase labelling. We used ordinary differential equation models to describe the fluxes of cells between the BrdU$^{+/-}$ Ki67$^{\text{high/low}}$ populations within the CD4 T$_{\text{CM}}$ and T$_{\text{EM}}$ subsets. Prior distributions on the rates of flow of new cells into each subset (θ) were chosen to be the posterior distributions on θ obtained from modelling the accumulation of donor cells in busulfan chimeric mice, as described in Text C. We assumed that kinetic heterogeneity within each subset could be represented with two subpopulations with distinct rates of division and death. In the branched model, these subpopulations (denoted A and B) are fed separately from the same precursor, at total rate θ, in unknown proportions. The linear model assumes constant flow from source into population A, which then transitions into population B at per capita rate γ. The burst model also assumes that the precursors enter population A, and cells transition into population B at per capita rate γ, and re-enter population A upon division. In all models, cells from from the precursor population are assumed to be Ki67^{high}, based on the assumption that new memory T cells have recently undergone clonal expansion.

Levels of Ki67 protein decline continuously after mitosis but, as is standard in flow cytometry analysis, cells are categorized into binary states Ki67^{high} and Ki67^{low}. We therefore modelled the transition between these two states using intermediate Ki67^{high} compartments, such that the residence time within the Ki67^{high} compartment is gamma-distributed. During the labelling phase, the delay before BrdU^{+}Ki67^{low} cells appeared was clearly apparent, suggesting that the variance in the residence time within Ki67^{high} was small. To describe this kinetic we used 10 intermediate Ki67^{high} states. We also used two intermediate BrdU compartments, reflecting the assumption that in the absence of label, BrdU^{+} cells become BrdU^{-} after two divisions. We also assumed that in the delabelling phase the BrdU content of the source population declined exponentially at a rate μ, which was also estimated.

The following equations describe the BrdU labelling and de-labelling phases for linear and branched models. For the branched model, the parameter F_A denotes the fraction of the influx that enters subset A, while γ (the rate at which type A cells differentiate into type B) was set to zero. For the linear and burst models, γ was a free parameter, while F_A was set to one. The only structural difference between the linear and burst models is that in the burst model, division of type B results in a transition back to type A.

Labelling phase:

$$\frac{d}{dt}A_{\hat{k}}^{\hat{b}} = F_A\theta - (\delta_A + \gamma + \hat{k}\beta + \alpha_A)A_{\hat{k}}^{\hat{b}} + 2\epsilon\alpha_A\sum_{i=0}^{\hat{b}}\sum_{j=0}^{\hat{k}}A_j^i$$

$$\frac{d}{dt}A_{\hat{k}}^{i} = 2(1-\epsilon)\alpha_A\sum_{j=0}^{\hat{k}}A_j^{i+1} - (\delta_A + \gamma + \alpha_A + \hat{k}\beta)A_{\hat{k}}^{i} \qquad \text{for } 1 \le i < \hat{b}$$

$$\frac{d}{dt}A_{\hat{k}}^{0} = 2(1-\epsilon)\alpha_A\sum_{i=0}^{1}\sum_{j=0}^{\hat{k}}A_j^{i} - (\delta_A + \gamma + \alpha_A + \hat{k}\beta)A_{\hat{k}}^{0}$$

$$\frac{d}{dt}A_j^i = \hat{k}\beta A_{j+1}^i - (\delta_A + \gamma + \alpha_A + \hat{k}\beta)A_j^i \qquad \text{for } 0 \le i \le \hat{b} \text{ and and } 1 \le j < \hat{k}$$

$$\frac{d}{dt}A_0^i = \hat{k}\beta A_1^i - (\delta_A + \gamma + \alpha_A)A_0^i \qquad \text{for } 0 \le i \le \hat{b}$$

$$\frac{d}{dt}B_{\hat{k}}^{\hat{b}} = (1-F_A)\theta + \gamma A_{\hat{k}}^{\hat{b}} - (\delta_B + \hat{k}\beta + \alpha_B)B_{\hat{k}}^{\hat{b}} + 2\epsilon\alpha_B\sum_{i=0}^{\hat{b}}\sum_{j=0}^{\hat{k}}B_j^i$$

$$\frac{d}{dt}B_{\hat{k}}^{i} = \gamma A_{\hat{k}}^i + 2(1-\epsilon)\alpha_B\sum_{j=0}^{\hat{k}}B_j^{i+1} - (\delta_B + \alpha_B + \hat{k}\beta)B_{\hat{k}}^{i} \qquad \text{for } 1 \le i < \hat{b}$$

$$\frac{d}{dt}B_{\hat{k}}^{0} = \gamma A_{\hat{k}}^0 + 2(1-\epsilon)\alpha_B\sum_{i=0}^{1}\sum_{j=0}^{\hat{k}}B_j^{i} - (\delta_B + \alpha_B + \hat{k}\beta)B_{\hat{k}}^{0}$$

$$\frac{d}{dt}B_j^i = \gamma A_j^i + \hat{k}\beta B_{j+1}^i - (\delta_B + \alpha_B + \hat{k}\beta)B_j^i \qquad \text{for } 0 \le i \le \hat{b} \text{ and } 1 \le j < \hat{k}$$

$$\frac{d}{dt}B_0^i = \gamma A_0^i + \hat{k}\beta B_1^i - (\delta_B + \alpha_B)B_0^i \qquad \text{for } 0 \le i \le \hat{b}$$

De-labelling phase:

$$\frac{d}{dt}A_{\hat{k}}^{\hat{b}} = F_A\theta e^{-\mu t} - (\delta_A + \gamma + \hat{k}\beta + \alpha_A)A_{\hat{k}}^{\hat{b}}$$

$$\frac{d}{dt}A_{\hat{k}}^{i} = 2\alpha_A\sum_{j=0}^{\hat{k}}A_j^{i+1} - (\delta_A + \gamma + \alpha_A + \hat{k}\beta)A_{\hat{k}}^{i} \qquad \text{for } 1 \le i < \hat{b}$$

$$\frac{d}{dt}A_{\hat{k}}^{0} = F_A\theta(1-e^{-\mu t}) + 2\alpha_A\sum_{i=0}^{1}\sum_{j=0}^{\hat{k}}A_j^{i} - (\delta_A + \gamma + \alpha_A + \hat{k}\beta)A_{\hat{k}}^{0}$$

$$\frac{d}{dt}A_j^i = \hat{k}\beta A_{j+1}^i - (\delta_A + \gamma + \alpha_A + \hat{k}\beta)A_j^i \qquad \text{for } 0 \le i \le \hat{b} \text{ and } 1 \le j < \hat{k}$$

$$\frac{d}{dt}A_0^i = \hat{k}\beta A_1^i - (\delta_A + \gamma + \alpha_A)A_0^i \qquad \text{for } 0 \le i \le \hat{b}$$

$$\frac{d}{dt}B_{\hat{k}}^{\hat{b}} = (1-F_A)\theta e^{-\mu t} + \gamma A_{\hat{k}}^{\hat{b}} - (\delta_B + \hat{k}\beta + \alpha_B)B_{\hat{k}}^{\hat{b}}$$

$$\frac{d}{dt}B_{\hat{k}}^{i} = \gamma A_{\hat{k}}^i + 2\alpha_B\sum_{j=0}^{\hat{k}}B_j^{i+1} - (\delta_B + \alpha_B + \hat{k}\beta)B_{\hat{k}}^{i} \qquad \text{for } 1 \le i < \hat{b}$$

$$\frac{d}{dt}B_{\hat{k}}^{0} = (1-F_A)\theta(1-e^{-\mu t}) + \gamma A_{\hat{k}}^0 - (\delta_B + \alpha_B + \hat{k}\beta)B_{\hat{k}}^{0} + +2\alpha_B\sum_{i=0}^{1}\sum_{j=0}^{\hat{k}}B_j^{i}$$

$$\frac{d}{dt}B_j^i = \gamma A_j^i + \hat{k}\beta B_{j+1}^i - (\delta_B + \alpha_B + \hat{k}\beta)B_j^i \qquad \text{for } 0 \le i \le \hat{b} \text{ and } 1 \le j < \hat{k}$$

$$\frac{d}{dt}B_0^i = \gamma A_0^i + \hat{k}\beta B_1^i - (\delta_B + \alpha_B)B_0^i \qquad \text{for } 0 \le i \le \hat{b}$$

Text B Model fitting

Initialisation Motivated by the experimental observations that cells numbers and Ki67 expression levels were approximately constant over the labelling assay, we assumed that each memory population (T_{CM} and T_{EM}, host/donor) was in (quasi-) equilibrium. To establish initial conditions for the model for each sampled set of parameters, we set all BrdU-labelled species to zero, and used the rk45 ODE solver to 10000 days to allow the system to attain steady state. This initialisation step determined the initial sizes of all species prior to BrdU administration, for each set of model parameters.

Modelling noise/mouse-to-mouse variation The model yields total cell numbers and the proportions of cells in the four quadrants of BrdU$^{\pm}$ Ki67$^{\pm}$. We assumed that uncertainty in the total numbers was lognormally distributed, and used a Dirichlet multinomial model to describe the assignment of cells to each of the four quadrants. The Dirichlet multinomial distribution is a discrete multivariate distribution for k variables $x_1 \ldots x_k$ where each $x_i \in (0, 1)$. Uncertainty in the assignment of cells to quadrants – which principally reflects variation between mice in the frequencies of cells in each quadrant – was described with an overdispersion parameter ϕ with an exponential prior.

Definition of priors For consistency of interpretation, we defined the subsets' loss rates (δ_A and δ_B) and division rates (α_A and α_B) such that the rates defining subset A were greater than those defining B. These rate constants were then sampled from a simplex that ensure all populations remained positive and finite in size. In addition each loss rate δ was constrained to be greater than the division rate to ensure the population remained at steady state with an influx of cells. The Ki67 lifetime $1/\beta$ was constrained to lie within 3-4 days, based on our previous studies. We chose broad priors for the rate of loss of BrdU within the source (μ) in the delabelling phase, and the efficiency of BrdU uptake per cell division during the labelling phase (ϵ). Details of priors are given at github.com/elisebullock/tcellmemorypaper.

Text C Estimating the rates of constitutive influx into CD4 T_{CM} and T_{EM}

Within busulfan chimeric mice, the accumulation of donor-derived T_{CM} or T_{EM} (Fig C) was reasonably well described by a simple model with a single average rate of turnover, λ;

$$\text{Donor cells} \quad \frac{dD}{dt} = f_d\,\theta - \lambda D; \qquad \text{Host cells} \quad \frac{dH}{dt} = (1 - f_d)\theta - \lambda H, \tag{1}$$

where f_d is the proportion of the constitutive influx of memory θ that comprises donor cells, and we model from the time at which donor memory T cells start to appear in lymph nodes. For each subset (T_{CM} and T_{EM}) and in young and old mice, we fitted this model simultaneously to the steady state numbers $N = D + H = \theta/\lambda$ and the chimerism timecourse $D(t)/N$ (Fig C), using data from the chimeric mice we studied here augmented with data from age-matched chimeric mice aggregated from other experiments. This procedure yielded posterior distributions of the total (host plus donor) influx rates θ (Table A) that were only weakly correlated with those of the other parameters (N, λ, and f_d).

Text D Explaining the progressive loss of Ki67 in donor cells with mouse age and the convergence of host and donor cell dynamics in older mice

To understand the patterns of host/donor cell differences at the level of their population dynamics, we derived an expression for the proportion of a memory population that is fast cells, p_A, in the branched model:

$$p_A^{\text{branched}} = \frac{1}{1 + \left(\frac{1-f_A}{f_A}\right) \lambda_A/\lambda_B}, \tag{2}$$

where f_A is the proportion of the source entering the fast population, which was slightly lower for $\mathrm{T_{EM}}$ than $\mathrm{T_{CM}}$ (Fig 4 in the main text). For both subsets we saw no significant differences in f_A between host and donor cells or between young and old mice, indicating that for each subset the rationing of new memory cells into fast and slow subsets was an invariant quantity. The differences in the donor/host fast fractions must therefore derive from the ratio of the loss rates of fast to slow populations, λ_A/λ_B. In young mice, fast donor $\mathrm{T_{CM}}$ clones were marginally less persistent than host counterparts ($\lambda_A^{\text{donor}} \gtrapprox \lambda_A^{\text{host}}$) but slow donor clones were much less persistent than slow host clones ($\lambda_B^{\text{donor}} > \lambda_B^{\text{host}}$). Thus, for $\mathrm{T_{CM}}$, λ_A/λ_B was smaller for donor than host, leading to a greater fast cell fraction and so explaining the donor-skewed Ki67 fraction (Fig 2D in the main text). For $\mathrm{T_{EM}}$ the picture was similar; in the younger cohort, clonal lifespans of fast cells were similar for donor and host, but slow donor clones were substantially shorter lived than host clones. Therefore, again, λ_A/λ_B was smaller for donor than host. In older mice, all differences in parameters between host and donor cells shrank, although the fast fraction remained significantly higher for donor in both $\mathrm{T_{CM}}$ and $\mathrm{T_{EM}}$. These patterns reflected the degree of convergence of Ki67 expression of host and donor cells (Fig 2D).

These conclusions also held for the linear and burst models. For both, the fraction of the population made up of fast cells is given by the simpler expression

$$p_A^{\text{linear/burst}} = \frac{1}{1 + \gamma/\lambda_B}, \tag{3}$$

where γ is the rate of differentiation from fast (A) to slow (B). For the linear model, for both $\mathrm{T_{CM}}$ and $\mathrm{T_{EM}}$, we saw no differences in γ between host and donor, or across mouse age (Fig G). In contrast, λ_B, the loss rate of slow clones, was greater for donor than for host in the younger mice, again explaining the higher fast fraction among donor cells in young mice, and hence their higher Ki67 expression. In older mice, λ_B for donor cells approached that of host, yielding the same conclusion drawn from the branched model. For the burst model, the same trends held for λ_B (Fig I). There was an additional contribution from γ, the rate of return of fast cells to a more quiescent state. In younger mice, this rate was estimated to be slightly lower for donor than for host cells (that is, younger memory cells 'burst' for slightly longer on average than older cells); this difference also acted to increase the fast fraction p_A among donor cells.

Text E Calculation of mean lifespan in the branched model

Suppose the donor fraction is f_D, the fast cell fractions within host and donor are f_A^H and f_A^D, respectively, and δ_A^D and δ_B^D are the loss rates of fast and slow donor cells, respectively, with correspondingly δ_A^H and δ_B^H for host cells. Then the population-average loss rate $\bar{\delta}$ is given by

$$\begin{aligned} \bar{\delta} &= f_D\bar{\delta}_D + (1-f_D)\bar{\delta}_H, \\ \text{where } \bar{\delta}_D &= f_A^D\delta_A^D + (1-f_A^D)\delta_B^D \text{ and } \bar{\delta}_H = f_A^H\delta_A^H + (1-f_A^H)\delta_B^H. \end{aligned}$$

The mean lifespan is then $1/\bar{\delta}$.

Text F Obtaining mean lifespans through estimating the total production rate

We present two methods of obtaining an approximate average loss rate (and hence mean lifespan) by measuring the total rate of production. These are equal for a population at equilibrium.

The first is to use information contained in the initial growth in the BrdU labelled fraction. De Boer and Perelson [1] used data from Younes *et al.* [2] who found that 35% and 60% of memory CD4 T cells were BrdU$^+$ after 3 and 10 days of labelling, respectively, to estimate an expected life span of between 14 and 22 days. To generalise their modelling approach, consider a memory population of size N at equilibrium, fed from a source at rate θ, dividing at average rate α, and lost at rate δ. During BrdU administration, assume the source is entirely labelled, and unlabelled memory cells take up BrdU with probability ϵ per division. If we denote unlabelled cells N_U and labelled cells N_L, then

$$\frac{dN_U}{dt} = -(\epsilon\alpha+\delta)N_U + (1-\epsilon)\alpha N_U, \quad \frac{dN_L}{dt} = \theta + 2\epsilon\alpha N_U + (\alpha-\delta)N_L. \tag{4}$$

The labelled fraction $L = N_L/N$ is given by

$$\frac{dL}{dt} = \frac{1}{N}\frac{dN_L}{dt} = (\delta - \alpha + 2\epsilon\alpha)(1-L), \tag{5}$$

where we have used $N_U = N - N_L$, and the steady state condition $dN/dt = 0$ yielding $\theta/N = \delta - \alpha$. The initial upslope of the labelled fraction L is then $p = \delta - \alpha + 2\epsilon\alpha$. If the source is entirely unlabelled,

$$\frac{dL}{dt} = 2\epsilon\alpha - L(2\epsilon\alpha - \alpha + \delta), \tag{6}$$

and so $p = 2\epsilon\alpha$. Therefore, depending on the rate of influx and its label content, the initial upslope in the BrdU$^+$ fraction can be a combination of both the average division and loss rates, as well as the uptake efficiency.

De Boer and Perelson's calculation assumed that memory CD4 T cells are entirely self-renewing, such that $\theta = 0$ and $\alpha = \delta$, and that uptake of BrdU by dividing cells is 100% efficient. Eq 6 then yields $dL/dt = 2\delta(1-L)$, which they fitted to the Younes data to obtain the average lifespan $1/\delta$. The self-renewing approximation is reasonable – we estimate that the influxes into T_{CM} and T_{EM} are of the order 1% of the population size per day, so we can proceed by neglecting them. However we established that the BrdU uptake efficiency ϵ is considerably less than 1, and was well constrained by the steepness of growth in the BrdU$^+$Ki67^{high} fraction ($\epsilon = 0.55$ in younger cohort, and 0.75 in the older cohort, which were separate experiments). The mean lifespan is then reduced to $2\epsilon/p$, as quoted in the main text.

The second approach is to derive the average rate of turnover using the frequency of cells expressing Ki67. Consider again the memory population at steady state size N with cells entering at rate θ, dividing at an average rate α and dying at average rate δ:

$$\frac{dN}{dt} = \theta + (\alpha - \delta)N = 0 \implies \alpha N + \theta = \delta N. \tag{7}$$

We know that Ki67 is expressed for a time $T \simeq 3$ days, and we assume that all cells entering memory are Ki67^{high}. Then the number of cells in the population at any time t that are Ki67^{high}, K^+, is equal to the number that entered or divided in the last T days, and survived to time t:

$$K^+ = \int_{t-T}^{t} (2\alpha N + \theta)\, e^{-\delta(T-s)} ds = \frac{2\alpha N + \theta}{\delta}(1 - e^{-\delta T}) = N\left(1 + \frac{\alpha}{\delta}\right)(1 - e^{-\delta T}). \tag{8}$$

If the influx per unit time makes up a small proportion of the population ($\theta \ll N$), as we found for both T_{CM} and T_{EM}, then $\theta \simeq 0$ and $\alpha \simeq \delta$. Then using Eq 7, the Ki67^{high} proportion $k = K^+/N \simeq 2(1 - e^{-\delta T})$. The mean lifespan $\tau = 1/\delta$ is then

$$\tau = \frac{-T}{\ln(1 - k/2)} \simeq \frac{2T}{k} \text{ when } k \text{ is small.} \tag{9}$$

Text G Predicting patterns of Ki67 expression among YFP$^+$ and YFP$^-$ cells following adoptive transfer

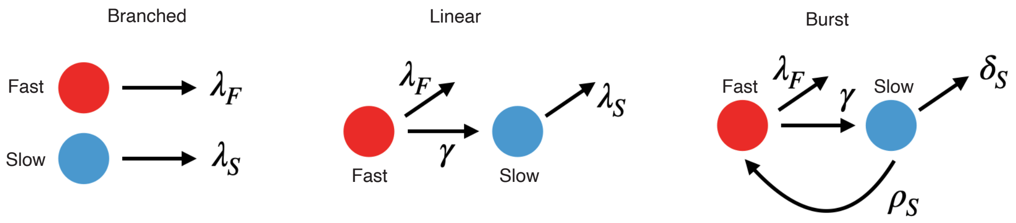

Figure A: Schematic of the three models, indicating the key parameters.

We can use each model to predict the outcome of transferring fast and slow cells to a congenic host after labelling Ki67-expressing cells with YFP. First, we note that in all models, we estimate that fast clones are lost more rapidly than slow ($\lambda_F > \lambda_S$).

The branched model predicts that slow cells, which are largely YFP$^-$ but will nevertheless contain some YFP$^+$ cells, will come to dominate over time. Therefore, Ki67 in both YFP$^+$ and YFP$^-$ populations will fall, and converge.

In the linear model, the slow cells will comprise cells that were slow upon transfer, and cells that subsequently transitioned from fast. Since $\lambda_F > \lambda_S$, selection will occur within the slow population for cells that were originally slow. So again, Ki67 overall falls to a lower level, that is independent of YFP expression.

Finally, in the burst model, there is a period just after transfer when YFP$^+$ cells are almost all transiently fast, and Ki67^{high}. Without new fast cells flowing in, the system will then settle to a lower level of Ki67 expression. YFP$^+$ and YFP$^-$ cells then behave identically, because all have the potential to re-enter the burst phase at random.

In summary, all three models predict the outcome of the transfer experiment.

Text H Predicting the timecourses of mTomato expression in CD4 reporter mice

We generated predicted timecourses of mTom-expressing cell frequencies as follows. First, we assumed that naive CD4 T cells were the dominant precursor of T_{CM}. We then fitted a phenomenological power-law function to the timecourse of $mTom^{+}$ cells starting at day 8 after tamoxifen treatment, at which point mTom expression was maximal in all compartments (Fig 7C in the main text). This function defined the time-dependent source population for $mTom^{+}$ CD4 T_{CM}. We assumed that $mTom^{+}$ and $mTom^{-}$ cells transitioned from CD4 naive to T_{CM} at the same rates. Second, we initialized the populations of Ki67-high, intermediate and low CD4 T_{CM} and T_{EM} within $mTom^{+}$ and $mTom^{-}$ cells, using the observed overall Ki67 fraction; and distributed these between fast and slow cells using the fast fraction derived from our fitted parameters (Text D). This process yielded the initial numbers of cells within all subpopulations (Text A). Then, using the parameters derived from fitting the BrdU/Ki67 timecourses, and the function describing the $mTom^{+}$ fraction among naive CD4 T cells, we simulated 150-day time courses of the $mTom^{+}$ fractions within T_{CM} and T_{EM} (Fig 7D in the main text), considering both naive or T_{CM} as possible sources for T_{EM}.

References

1. De Boer RJ, Perelson AS. Quantifying T lymphocyte turnover. Journal of Theoretical Biology. 2013;327:45–87.
2. Younes SA, Punkosdy G, Caucheteux S, Chen T, Grossman Z, Paul WE. Memory phenotype CD4 T cells undergoing rapid, nonburst-like, cytokine-driven proliferation can be distinguished from antigen-experienced memory cells. PLoS Biology. 2011;9(10):e1001171.
