# Supplementary material for: The dynamics and longevity of circulating CD4+ memory T cells depend on cell age and not the chronological age of the host
Source: PLoS Biol. 2024 Aug 13;22(8):e3002380. doi: 10.1371/journal.pbio.3002380 (PMC11321570; doi:10.1371/journal.pbio.3002380)
Supplement: Attachment — Submitted filename: Bullock Plos Biology 2024 - Response 1.pdf [file pbio.3002380.s003.pdf]

Response to reviewers, Jan 2024
Bullock et al., "Cell age, not chronological age, governs the dynamics and longevity of circulating CD4 memory T cells".

We're very grateful to all three reviewers for their insightful and helpful critiques.

Rev. 1:
In this article, Bullock et al have combined wet lab experiments with mathematical modelling to examine memory phenotype CD4 T cells in mice. The field struggles to classify these cells that likely are composed of different populations that have responded following stimulation with cytokines and/or antigens, and in some cases are specific for antigens which are presented for short periods of time or specific for antigens which persist. The authors dissect the population of memory CD4 T cells in various ways - first by whether they express the lymph node homing molecule, CD62L and are classified by the field as Tcentral memory (CD62L+) or Teffector memory (CD62Llo/negative). Second, by whether they express a congenic molecule that marks them as host derived, or derived from a transfer of bone marrow stem cells when the hosts are around 50-100 days old. The authors also follow on from previous findings in the field describing that some memory phenotype CD4 T cells proliferate frequency and some very infrequently and there is not currently a consensus on an underlying mechanism to explain these different behaviours.

The authors' aims were to understand the proliferation of the CD4 T cells within these populations, predict life spans, and investigate the relationship between the memory populations.

The main findings described in the manuscript are:
1. Fast and slow dividing memory CD4 T cells can be found in both Tcm and Tem populations and in young and older mice.
2. But memory cells are more likely to divide in younger than older mice and at both time points, the donor cells are more likely to divide than the original host memory cells.
3. The differences in proliferation kinetics are less pronounced in the older mice than the younger mice.
4. The formation of Tem and Tcm cells can be explained by two different models in which Tcm and Tem are separate populations (branching) or in which Tcm convert into Tem (linear).
5. The life span of memory cell populations is similar to previous calculated values from other investigators.

The authors use these findings to conclude that cell age, rather than host environment, predict the proliferate behaviour of memory phenotype CD4 T cells. These data are potentially important as the longevity of memory CD4 T cells is key to understanding protective immunity to pathogens that the host as met previously through infection or vaccination. Long lived populations of CD4 T cells are more likely to provide durable immunity than short lived cells.

Overall, this is a well-written manuscript that combines the strengths of the authors in wet lab studies and mathematical modelling. I am a wet lab biologists and have limited ability to review the veracity of the modelling aspects of the manuscript.

The main strengths are that the authors have generated a substantial data set that allows for analysis of populations of memory CD4 T cells across time and which can probe the proliferation dynamics of the cells and generated different models to test hypotheses about the relationships between the cells.

The main weaknesses are that the authors have made a number of assumptions as they moved from findings to conclusions (see points 1 and 2 below) and that much of the work confirms previous findings (lifespan of memory populations) rather than presenting novel conclusions or hypotheses (i.e. differentiation of naïve cells into memory cells that may/may not require cells to go through a Tcm before a Tem phenotype).

Main concerns:

1. Are the authors modelling different processes between host and donor cells?
The diagram in Fig1A suggests that naïve CD4 T cells are predominantly donor derived at the analysis time point; the data in Fig2D suggests that donor derived memory phenotype cells are more likely to be Ki67+ than host memory cells. Is the explanation for the latter that the cells are coming from the naïve population? The authors investigate this later in the manuscript and conclude that, at least the Tcm population, is likely derived from the naïve CD4 T cell pool. They state in the discussion (line 419) that there is 'substantial recruitment of host cells into memory' and imply these are coming from the naïve pool. It wasn't clear to me what data this referred to and how the author ruled out that the host proliferating memory cells were memory cells re-activated by antigen and/or cytokine while the donor cells where newly activated naïve cells entering the activated/memory pool for the first time.

Thus, it is not clear to me how the authors can argue that they are comparing 'older' host cells with 'younger' donor cells rather than 'memory' with 'naïve' T cells. If they can't make this distinction, their main conclusion about the distinctions in proliferative behaviour between young and old cells is not supported. In the older cohort of mice, the differences between the host and donor cells is much less - this may be because here they are mainly comparing memory host cells with memory donor cells.

Very early after BMT, you are indeed correct that all donor cells within the the CD44hi populations (i.e. CM and EM) will be very recently generated memory cells - fresh from their precursor population, which we argue is dominantly the naive pool for CM, and the CM pool for EM. However, this is not the case in the mouse cohorts we model here, which even though they are labeled 'young' and 'old', are both many weeks post-BMT.

There are two key points here; first, we show that influx into both CD4 CM and EM pools represents about 1-2% of the population size per day, so our description of influx as `substantial' is misplaced (although it persists throughout life). Thank you for pointing this out - we replace it with "continued". Second, both donor AND host cells continue to enter into memory after BMT, in comparable numbers, because substantial number of host memory precursors remain (see Figure 6 for the measured chimerism of naive CD4 T cells in the two cohorts). So the large differences in Ki67 expression between host and donor cells in the young mice cannot be explained purely by the behavior of small numbers of very recent (and exclusively donor-derived)

immigrants. Indeed, over the timescales we are modeling here, even in the younger mice, the donor population will include cells that have resided in memory for many weeks and so will behave as established memory cells.

Instead, the modeling shows that the Ki67 difference can be explained very simply by progressive declines in average proliferation rates with the time spent in memory. That is, the fast/slow composition of a memory cohort shifts towards slow (less proliferative) cells over timescales of weeks. The host/donor difference is quite pronounced in the young mice, because the donor cells on average are younger than host cells, and hence enriched for fast cells. But crucially, in the older animals, host and donor cells look very similar – because their age profiles (and hence fast/slow compositions) have converged. We would not see this behavior if the host/donor difference was as stark as you suggest. Another way to see this is in the shape of the BrdU labelling curves. The donor curve would look very different from the host curve if all proliferation derived from the small number of donor cells that had only just entered memory.

Your question motivated us to perform and analyse some new experiments to validate the idea that donor and host cells are really not intrinsically different in these busulfan chimeric mice (new section in Results, Figure 7).

2. Argument that 'cell-age' effects dominate over the cell's environment.
This follows on from the concern above but contains a further distinction that the authors have not considered. This is that while the donor and host CD4 T cells are within the same host, they may be found in distinct microenvironments in the secondary lymphoid organs, and thus exposed to different levels of cytokines, different types of antigen presenting cells, and thus more or less likely to be exposed to antigen.

This is an excellent point — these slow changes in memory kinetics might well derive from changes in circulation patterns. Both arguments could cause delineations in kinetics of host and donor cells. So spatial location, which would be related to e.g. receptor expression, would be a proxy for cell age and the availability of niche-dependent survival signals. Further spatial study of lymph nodes would be required to validate this theory. We hadn't posited a cell aging mechanism in the paper and so we now add this as a possibility in the Discussion.

3. Lifespans - the authors describe half-lives (35-140 days) and mean lifespan (18-20 days). Can they explain the different measurements?

Thanks for pointing this out - we have tried to clarify in the paragraph around line 200.

The difference derives from the concept of the persistence of population that is both being lost and undergoing self renewal, and the lifespan of the constituent cells. For example, one might imagine a population of cells dividing and dying over time frames of days, but these two rates might be sufficiently balanced that the population is a whole could persist for weeks or months. To distinguish this timescale from cell lifespan, we refer to it as 'clonal lifespan'. It applies in fact to any dynamic population of cells, clonal or not; but the name is motivated by the idea that self renewal would preserve TCR specificity, and so this concept of persistence is highly relevant for measuring the persistence of cohorts of TCR clones.

4. The authors have limited discussion on the cells that included/excluded from the populations. They have excluded CD25+ cells and thus will be excluding some, but not all of the Tregs. While CD25 exclusion will also mean they are excluding recently activated T cells from the analysis, CD25 is very transient on activated cells and thus their 'memory' populations are likely to include 'activated' cells. The authors themselves state that memory phenotype cells are derived from naïve cells and that some of these may be responding to environmental antigens even without overt infection/vaccination. It would be helpful if the authors include a few statements to describe the likely composition of the cells they refer to as 'memory' cells.

The BrdU labelling experiments were undertaken several years ago now. The technical challenges around the relatively large staining panel for the BD-Fortessa, and combining methodologies for BrdU labelling and Ki67 staining (see our cited *Bioprotocol* papers), precluded inclusion of a Foxp3 stain. We shared your concern, and subsequently analysed Foxp3, CD25, and Ki67 expression by T cells from control mice. This confirmed the suspicion that CD25neg gated populations will not completely exclude Foxp3+ cells; approximately 10% of cells were Foxp3+. However, we found that using a CD25neg rather than a Foxp3- gate for conventional memory T cells had very little impact on our estimates of Ki67 expression frequencies within TCM and TEM (see below), this was the only pertinent information used for fitting.

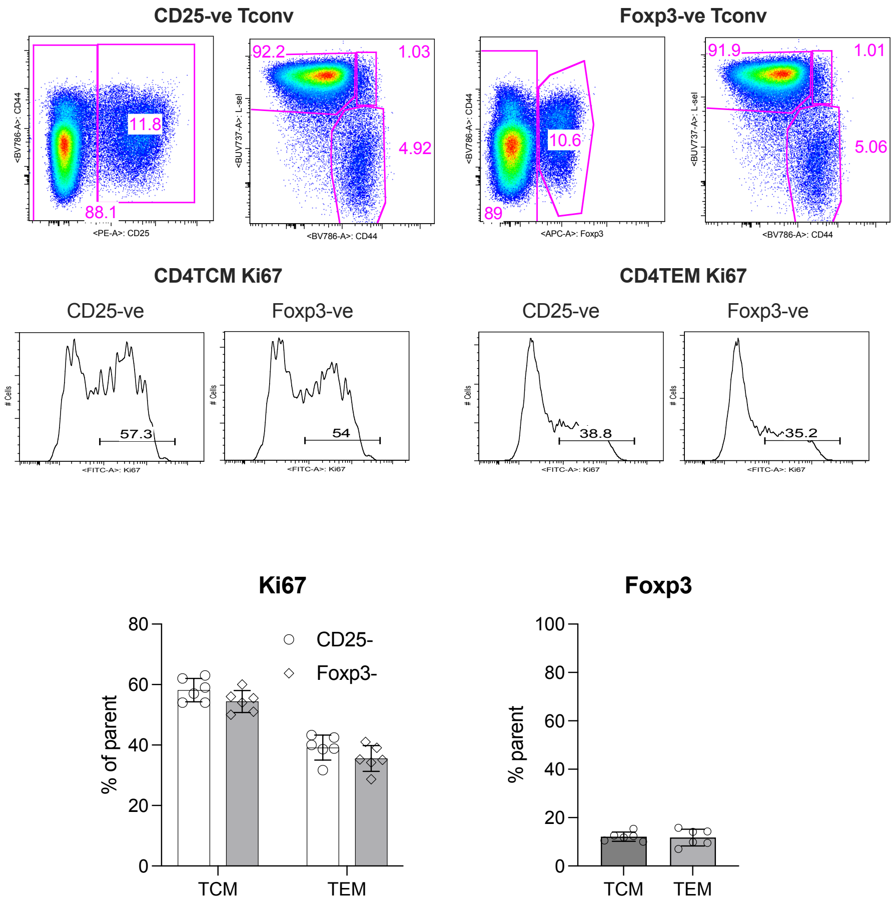

Minor concerns:
1. In the abstract, the author refer to clones - they have examined populations of cells not individual clones so this language is perhaps not appropriate.

Thanks, yes. See our response above. We have clarified in the text.

2. It would be useful if the authors provide example gating for the T cells analysed, e.g. in a supplemental figure. It is also now standard to include the fluorochrome used in the axis label of the flow plot e.g. in Fig 1C and to include the axes numbers.

We now provide the complete gating strategy in Fig S1, presented as you suggest.

3. In Figure 1 part C Ki67/BrdU panels, it is unclear if the cells displayed are all T cells or the CD45.1 or CD45.2 cells. The legend suggests that are 'stratified' by donor/host but it wasn't clear which (or both) are shown.

Thanks for picking this up. We are showing donor cells here and have corrected this in the caption.

4. In the methods, the authors should state the age of the mice used for the donor bone marrow, are these the same age as the host animals? This is important and HSC can show effects of age.

All donor mice were sex- and age-matched at 8 wks. (now stated at L68)

5. In Fig3C, CD4 Tcm proportion graph, it looks like there is only one blue/donor point at day 15. I am guessing there two points are on top of each other? Can they nudge one point slightly to enable the reader to see both points.

Again, thanks for picking this up! We have clarified in the caption that there were two points per time point and that these are occasionally overlaid.

6. In the legend of Fig 3, it would be helpful to state that the lines show the predicted curves for the branched model, this is my understanding from the text in the paragraph with lines number 185-194.

We have clarified that it was the branched model.

7. Unclear what the word 'cohoxhich' means on line 295. Perhaps meant to say 'cohort in which'?
8. Extra 'was' in line 390.

Thank you … a quite spectacular typo. Corrected.

9. The methods should contain information on which lymph nodes were examined and how the lymph nodes were processed and counted.

We have added this information to the methods.

10. The methods should state how the busulfan was given to the mice, how many donor cells were transferred, how the donor cells were transferred and how the bone marrow was prepared (especially how depleted of B and T cells). Alternatively, If a prior publication contains detailed methods, this could be referenced in the methods section.

Yes - the first line of Methods refers to our *Bioprotocols* paper where all these details can be found.

In terms of scope for an 'Update Article' my understanding is that the manuscript follows on from Rane et al, PLOS Biology, 2018. The 2018 paper examines survival of naïve CD4 T cells while the new manuscript examines memory CD4 T cells. The two studies thus ask similar questions of distinct populations using some similar methods. Thus, the two studies are certainly related but the results do go beyond the findings in the 2018 paper.

Thanks - we agree. This somehow was imposed on us during submission. We've requested a change in the classification.

Rev. 2: Jose Borghans – note that this reviewer has signed her review
This paper elegantly tears apart the effects of the age of the host versus the age of the cell on the dynamics of circulating memory CD4+ T cells, and thereby gives important insights into the long-term maintenance of immunological memory. Generally speaking, the manuscript is clearly written, makes an important contribution to the field of immunological memory, is based on robust analyses, and in my opinion fits the scope of PLoS Biology.

I have a few questions and suggestions:
- I was wondering why the authors "only" report results for CD4 memory T cells. I would assume one could obtain information from both CD4 and CD8 memory T cells of the same mice, and if those analyses have been performed, it would make a lot of sense to me to report them alongside the results on CD4 T cells. It would be interesting to see if CD4 and CD8 memory cells show a similar behaviour.

Good question. Ideally we would have studied CD4 and CD8 in tandem but the flow panel we used did not include CD49d, which distinguishes CD8 CM and VM cells, which we were unaware of when the BrdU timecourses were generated. VM cells are known to be established early in life and are likely functionally and kinetically distinct from CM. We have now rectified this issue and will include both CD4 and CD8 memory in future studies.

- The authors propose that "CD4 memory populations become enriched for older clones that have a fitness advantage over newly generated ones." This is the opposite of what we proposed in Swain et al. (2022, Frontiers in Immunology, "Effect of cellular aging on memory T-cell homeostasis"). In the latter paper, we propose that cellular aging (defined as a declining cellular fitness, either due to reduced proliferation or increased cell death) may help to keep the memory T-cell pool diverse, despite competition between memory cells for shared proliferation and survival factors. What are the authors' thoughts on how a diverse memory T-cell pool can be maintained over time if in fact the oldest memories outcompete the new ones? How would that work in the natural situation where people are frequently exposed to new antigens? Wouldn't this hamper the build-up of a diverse memory pool and reduce the chance to make new memories? And in this light, of the two options that i) older cells become longer-lived, or ii) older cells are biased towards being longer-lived, wouldn't the second one be more advantageous, because it would at least still allow (a random selection of) newly acquired memory cells to compete their way into the repertoire? I would love to hear the authors' thoughts on this perhaps slightly philosophical/teleological discussion.

Thanks for raising this important issue and pointing out the oversight! We now refer to your paper in the Discussion.

We have a few thoughts here:

1. The cell age effect we find is echoed in Rustom's CD8 YFV paper in PLos Comp Bio, where they saw the loss rate of memory declining - consistent with clonal lifespan slowly extending with residence time. Also, as you mentioned, we and others have shown that naive T cells appear to increase in lifespan with cell age. So this increasing-persistence effect is possibly a general T cell phenomenon. We now mention this.

2. Your notion of cell age and ours are slightly different - yours is defined by division number whereas ours is a heritable residence time 'clock'. Both our definitions of age are positively correlated with the time a cell or its descendants reside in memory, but maybe we need to be careful when comparing conclusions. In your model, 'fast' cells will age faster than slow cells, which we found divide quite infrequently. It's possible that both 'residence-age' and 'division-age' effects operate.

3. You're right that fluxes into the memory pool will be very different in a natural environment - different specificities and priming conditions. It may also be the case that the injection of new specificities into memory falls with age as infections are cleared by pre-existing cells and perhaps the ability of aged naive T cells to generate new memory is impaired. In these settings, preserving older memory cells may be beneficial.

We've added a reference to your paper and discussion of these points.

- What is shown in this paper for memory T cells is very reminiscent of what (some of) these authors have previously shown for naive CD4 and CD8 T cells. Please discuss the similarities and differences relative to naive T cells in the general Discussion.

Yes - we now highlight this issue in the Discussion (see also our response to Reviewer 3).

- The authors interpret the behaviour of donor-derived cells in the busulfan-experiments as the behaviour of young cells. Is there a risk that these cells behave differently because they came from a different source, i.e. from a relatively empty bone marrow? In other words, do the authors think the observed differences between old (host-derived) and young (donor-derived) cells could be due to the specific model used, and if not, why not?

This is a good point and we've added new data to address this.

We've shown before that reconstitution of bone marrow is very rapid, the host/donor composition of thymocytes equilibrates by 6 weeks, and the size and cellularity of the thymus do not change during this time (Hogan PNAS 2015). Further, total numbers of naive and memory cells in WT and busulfan chimeric mice are indistinguishable (Gossel eLife 2017), as well as Ki67 levels, suggesting no perturbation of thymic output, nor of peripheral cell kinetics. With these results alone, we have confidence that host and donor cell differences are not due to ontogenic effects. However there is still the possibility that the donor BM somehow generates cells with intrinsically different kinetic parameters. To address this, we have added data from two different experimental systems (see new section in Results, and the new Figure 7). We used these systems (i) to show that the basic assumptions of the model used to describe memory T kinetics in the busulfan chimeras also holds in non-chimeric mice and (ii) to use fitted models from the chimeras to accurately predict memory cell behavior in bulk in a completely different system.

- Related to this: I am a bit confused that the decline in Ki67 expression with age is so specific for the T cells from donor-origin. Even if age effects are due to cellular age and not to the age of the host, I would have expected to see a reduction in Ki67 expression with age for both host- and donor-derived

cells. After all, the host-derived cells in the young mice were only about 10 weeks older than the new donor-derived cells.

Great observation, thank you! The model fitting indicates that this increase in survival and decline in Ki67 with residence time must saturate over weeks to a few months - the changes don't continue indefinitely. This explains the similarity of Ki67 within host cells in the two cohorts of mice. We've added text to explain this.

- I find the section on "Clonal age effects explain changes in CD4 Tcm and Tem dynamics with mouse age" a bit of a tough read; especially from the second paragraph onward the style is a bit too technical for a journal like PLoS Biology.

We agree, it was rather horrible to read. We've cut and simplified this section a lot and put all the details in the Supp Info.

Rev. 3: Jorg Goronzy – note that this reviewer has signed his review
The manuscript by Yates, Seddon and coauthors comes from an investigative team that has shaped the field of T cell dynamics. Here, they perform mathematical modelling of data from DNA labelling with BrdU and expression of Ki67 using a fatemapping system, which allows them to quantify the dynamics of both young and established circulating memory CD4+ T cell subsets within both young and old mice. BrdU labeling and Ki67 expression are standard procedures. The chimeric mouse model where hematopoietic stem cells are selectively depleted by low-dose busulfan while keeping peripheral lymphocytes intact was published in PNAS in 2015 by the authors and is generally accepted as a tool. The innovation comes from combining these approaches with mathematical modeling that needs to be evaluated by another reviewer wiho has this expertise.

The main results are that cell-age dominates host-age and that T cell clones become more quiescent the older they are and that are more long-lived and have a fitness advantage over newly generated ones. As a result, CD4 memory populations become enriched for older clones which may have functional consequences. While this phenomenon has been qualitatively described (and one would like to see more context discussion from previous literature such as the studies by Susan Swain et al), the current manuscript provides the first quantitative and dynamic assessment.

Thanks - yes, these functional effects are important. In the Discussion, we now describe the work by Susan Swain and others regarding cell age effects, and connect both their and our results to the recent study from Jose Borghans' group regarding the preservation of functional memory.

Additional findings of interests are that newly produced T CM derive largely from the naive CD4 T cell population in bulk and that there is a flux from T CM to T EM, more consistent with a linear than a branching model. Overall, data are convincing and supportive of the interpretation.

One limitation of the study is that it is unclear how far these results on phenotypically defined memory cells can be applied to conventional antigen-specific memory cells. As these and other authors have shown, memory cells in bulk divide more rapidly than antigen-specific memory cells, possibly because antigen-specific T cells masquerade as naive cells or the dynamics of virtual memory cells may be different from antigen-specific cells. In their discussion, the authors discuss their data in the context of published data on antigen-specific cells, however, the limitations could be expressed more clearly.

Thanks, this is important. We now argue more clearly in the Discussion that the difference in division rates of MP and conventional antigen-specific memory cells likely does not reflect a fundamental difference between them, but instead derives from observing the continued recruitment of new proliferative cells into the MP population. Any defined cohort of newly generated MP cells will progressively slow and settle into the dynamics characteristic of antigen specific memory, which presumably are not typically chronically stimulated. The new experiment that we have added at the end of the Results section supports this inference.

Moreover, the antigen-specific data are mostly from CD8 T cells which may behave differently than CD4 T cells.

Our comparison of MP and antigen specific cell dynamics were restricted to CD4 memory studies, but you're correct that most studies of memory dynamics relate to CD8 T cells. We do draw parallels between the two in a dedicated paragraph. We've also added a potential explanation of the result of Choo et al. (Rustom's analysis of data from Rafi Ahmed's lab) who found that CD8 memory divides at the same rate as LCMV-specific memory; they used an adoptive transfer approach that removed the source of any new MP CD8 T cells.
